# Identification and functional analysis of early gene expression induced by circadian light-resetting in *Drosophila*

**DOI:** 10.1186/s12864-015-1787-7

**Published:** 2015-08-01

**Authors:** Adeolu B. Adewoye, Charalambos P. Kyriacou, Eran Tauber

**Affiliations:** Department of Genetics, University of Leicester, University Road, Leicester, LE1 7RH UK

**Keywords:** Circadian clock, Transcriptome, Light entrainment, *Drosophila*, Microarrays, Chromatin remodelling, Gene expression, Circadian phase shift

## Abstract

**Background:**

The environmental light–dark cycle is the dominant cue that maintains 24-h biological rhythms in multicellular organisms. In *Drosophila*, light entrainment is mediated by the photosensitive protein CRYPTOCHROME, but the role and extent of transcription regulation in light resetting of the dipteran clock is yet unknown. Given the broad transcriptional changes in response to light previously identified in mammals, we have sought to analyse light-induced global transcriptional changes in the fly’s head by using Affymetrix microarrays. Flies were subjected to a 30-min light pulse during the early night (3 h after lights-off), a stimulus which causes a substantial phase delay of the circadian rhythm. We then analysed changes in gene expression 1 h after the light stimulus.

**Results:**

We identified 200 genes whose transcripts were significantly altered in response to the light pulse at a false discovery rate cut-off of 10 %. Analysis of these genes and their biological functions suggests the involvement of at least six biological processes in light-induced delay phase shifts of rhythmic activities. These processes include signalling, ion channel transport, receptor activity, synaptic organisation, signal transduction, and chromatin remodelling. Using RNAi, the expression of 22 genes was downregulated in the clock neurons, leading to significant effects on circadian output. For example, while continuous light normally causes arrhythmicity in wild-type flies, the knockdown of *Kr-h1*, *Nipped*-A, *Thor*, *nrv1*, *Nf1*, *CG11155* (ionotropic glutamate receptor), and *Fmr1* resulted in flies that were rhythmic, suggesting a disruption in the light input pathway to the clock.

**Conclusions:**

Our analysis provides a first insight into the early responsive genes that are activated by light and their contribution to light resetting of the *Drosophila* clock. The analysis suggests multiple domains and pathways that might be associated with light entrainment, including a mechanism that was represented by a light-activated set of chromatin remodelling genes.

**Electronic supplementary material:**

The online version of this article (doi:10.1186/s12864-015-1787-7) contains supplementary material, which is available to authorized users.

## Background

Daily physiological and behavioural rhythms are regulated by molecular transcriptional-translational feedback circuits collectively referred to as the circadian clock [1, 2]. The period of oscillation generated by the circadian pacemaker is species-specific and ranges between 23–26 h in most eukaryotes under constant darkness (DD) [3]. The pacemaker is entrained by environmental cues, predominantly light–dark cycles, in a process that aligns the ‘free-running’ endogenous period of the circadian oscillator to the 24-h diurnal cycle.

In the laboratory, the phenotypic impact of light has been extensively studied using light pulse experiments. In these experiments, the free-running locomotor rhythm that occurs during continuous darkness (DD) is monitored before and after the presentation of a brief light pulse [4]. The response to the light depends on the stimulus intensity, phase, and light sensitivity of the organism. It is measured by comparing the phase of the rhythm before and after the light stimulus (phase shift). Light stimuli during early night delay rhythmic activities, whereas light stimuli during late night advance rhythmic activities. Stimuli during the subjective day have little or no effect on phase shift.

In *Drosophila*, the light resetting of the clock is mediated primarily by CRYPTOCHROME (CRY), a blue-light photoreceptor [5, 6]. Upon light activation, CRY interacts with TIMELESS (TIM), another photosensitive protein, resulting in TIM degradation [7]. Other proteins, such as JETLAG (JET) [8, 9], Slimb [10], COP9 signalosome [11], and CULLIN-3 [12], have also been shown to regulate the accumulation of TIM. A recent study in Drosophila S2 cells also identified BRWD3, a WD40 protein, as an E3 ligase that is required for CRY degradation by the ubiquitin-proteasome system [13]. This simplified circuit suggests that light resetting of the clock is mediated by CRY and TIM. However, it is clear that many more genes are involved in this response, some of which may be activated by light at the transcriptional level [14].

A broad transcriptional response to light pulses has been shown in various studies [15–19]. In mammals, light is transmitted via the retinal ganglion cells to the rhythmic cells of the suprachiasmatic nucleus [20]. This mediates the release of glutamate that binds N-methyl-D-aspartate receptors, which increases calcium influx in the suprachiasmatic nucleus [21, 22]. Subsequent activation of MAPK results in the phosphorylation of CREB that binds to *CRE* elements within *per1* and *per2* promoters [23–25] to activate their transcription. Light-activated genes represent a broad range of functional classes, such as stress response, DNA repair [19], cell cycle [26], and various metabolic pathways (e.g. heme metabolism) [17, 19].

In *Drosophila*, microarray analysis has demonstrated a profound impact of light on gene expression, with large numbers of transcripts driven by a clock-independent system [27]. The activation of this subset of light-induced transcripts require the *no receptor potential A* (*norpA*) gene, which is involved in visual transduction [27]. However, little is known about the immediate or early transcriptional changes associated with light-induced phase shifts in *Drosophila*. Here, we aimed to identify genes whose expression is modulated by an early-night light pulse and to explore the role of candidate genes in circadian light photosensitivity.

## Results

### Differentially expressed genes induced by light

**Fig. 1:**
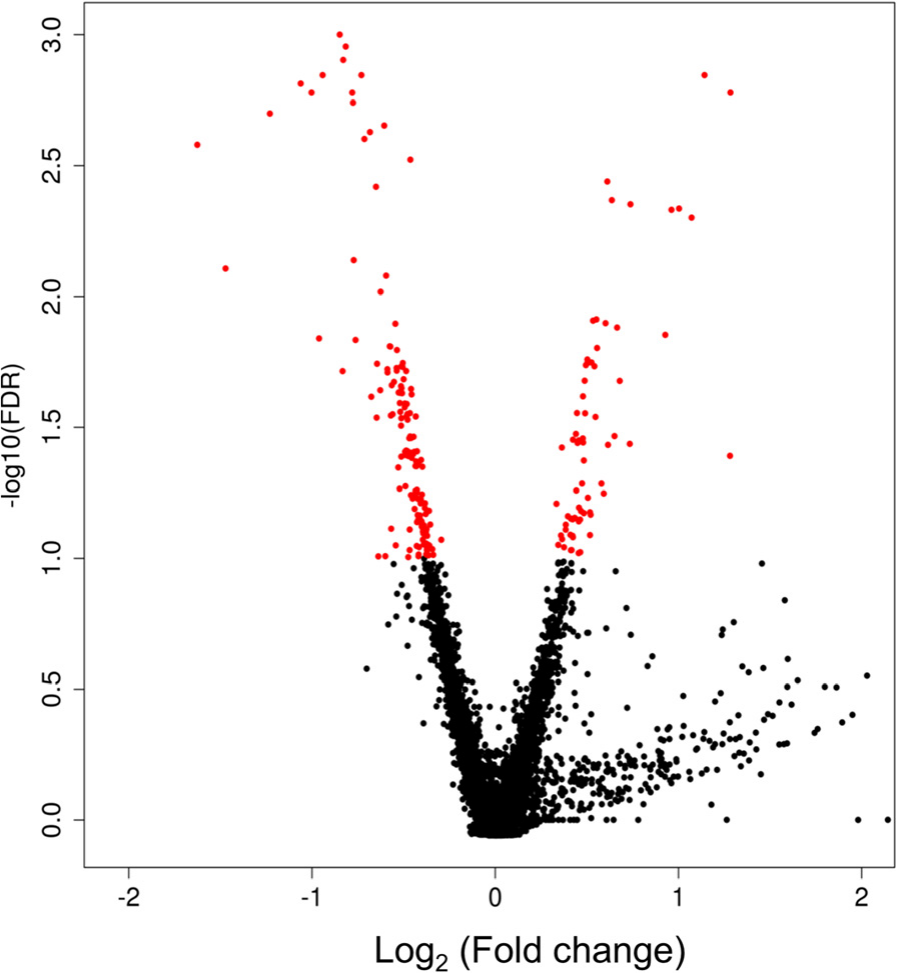
Identification of differentially expressed genes associated with early-night light response. Volcano plot showing the negative log_10_ of the false discovery rate value (Y axis) against log_2_ of the fold change (X-axis, light-pulse versus control samples). The differentially expressed genes at a false discovery rate < 0.1 are depicted in red

Analysis of the replicate microarrays within each condition (control and treated) showed that the intensity captured per probe across the whole chip was highly correlated (*r* = .98, *p* = ≤ .05, Additional file 1: Figure S1). As shown in Fig. 1 and Additional file 1: Table S1, we identified 200 (87 upregulated and 113 downregulated) differentially expressed genes (DEGs) that respond to light stimulation, at a false discovery rate threshold of 10 %. A subset of DEGs was randomly selected and tested by quantitative real-time PCR. Out of 16 genes, 11 (69 %) showed a significant difference and the same trend as in the microarray experiment (Additional file 1: Table S2). One of the DEGS is *period (per)*, a gene known to modulate circadian clock and its outputs. Our results showed that *per* is downregulated by light stimulation during early night. A similar rapid decrease (10–30 %) of *per* mRNA following a light pulse at Zeitgeber Time (ZT) 15 has been previously reported [28]. This observation further confirms the critical role of the transcriptional feedback loop in light-induced phase shifting of the clock.

### Gene ontology functional enrichment analysis

To explore the biological significance of each DEG, we used the clusterProfiler bioinformatics tool to analyse Gene Ontology (GO) category enrichment [29]. Using hypergeometric tests for enrichment at a *p* value < .05 and adjusted for multiple testing, the DEGs were classified into 416 functional groups consisting of 40 molecular functions, 41 cellular components, and 335 biological processes. Additional file 1: Table S3 provides a complete list of the supplementary material. We found the GO terms for biological processes to be significantly enriched in response to stimulus (GO: 0009605), whereas those for molecular functions were enriched in gated channel activity (GO: 0022836) and ion channel activity (GO: 0005216) (see Additional file 1: Figures S2 and S3). For the cellular components, GO terms related to extracellular region and plasma membrane were the most enriched (see Additional file 1: Figure S4), suggesting that most of the DEGs were active in these two cellular compartments. One of the biological processes that represents a high proportion of the DEGs is biological regulation (GO: 0065007) and consists of genes involved in gene regulation and chromatin remodelling. From this analysis, 22 genes listed in Table 1 were selected for behavioural characterisation.

**Table 1:** Genes selected for functional analysis

Gene	Fold change	FDR	biological function	Brain Enrichment^a^
DopR	−0.52	0.028	activation of adenylate cyclase activity	123
CG11155	−0.37	0.108	ion transport	12
sug	−0.82	0	positive and negative regulation of transcription	Eye
sif	−0.58	0.015	regulation of synapse structure and activity, synaptic transmission	25
Thor	−0.36	0.09	negative regulation of translational initiation, antibacterial humoral response	NA
Nf1	−0.51	0.022	locomotor rhythm, cAMP-mediated signalling, regulation of Ras protein signal transduction	17
pho	−0.39	0.075	negative regulation of gene expression	1.6
CalX	−0.38	0.077	Phototransduction	Eye
nrv1	−0.34	0.105	potassium ion transport, sodium ion transport	Eye
modifier of mdg4	−0.42	0.044	regulation of apoptosis, regulation of chromatin assembly or disassembly	2.6
Hr38	0.8	0	phagocytosis, engulfment	24
Fmr1	0.5	0.018	circadian rhythm, brain development, neurotransmitter secretion, synaptic transmission	4.3
CG7589	0.41	0.07	phagocytosis, engulfment	Eye
CG11597	0.6	0.013	protein amino acid dephosophorylation	2.1
CG2051^b^	0.44	0.055	histone acetylation; chromatin silencing at telomere	0.6
Nipped-A^b^	−0.46	0.034	Signalling, transcriptional co-activator. A key component of both the SAGA and Tip60 (NuA4) chromatin-modifying complexes.	1.9
trithorax^b^	−0.41	0.062	histone methylation; histone H3-K4 methylation	4.1
PSc^b^	0.48	0.035	chromatin remodelling	1.8
nejire^b^	−0.42	0.059	histone acetyltransferase activity, H3-K27 specific, H3-K18 specific	
Sirt6^b^	0.36	0.082	Predicated histone deacetylation activity, determination of adult life span	0.6
Kr-h1^b^	−0.37	0.089	transcription factor activity	2.3
Su(var)3-9^b^	0.48	0.024	Histone methyltransferase	2.3

^a^Enrichment of expression compared to whole body, data from FlyAtlas.2 [58]

^b^Genes associated with chromatin modifications

### Functional analysis of chromatin remodelling genes

**Fig. 2:**
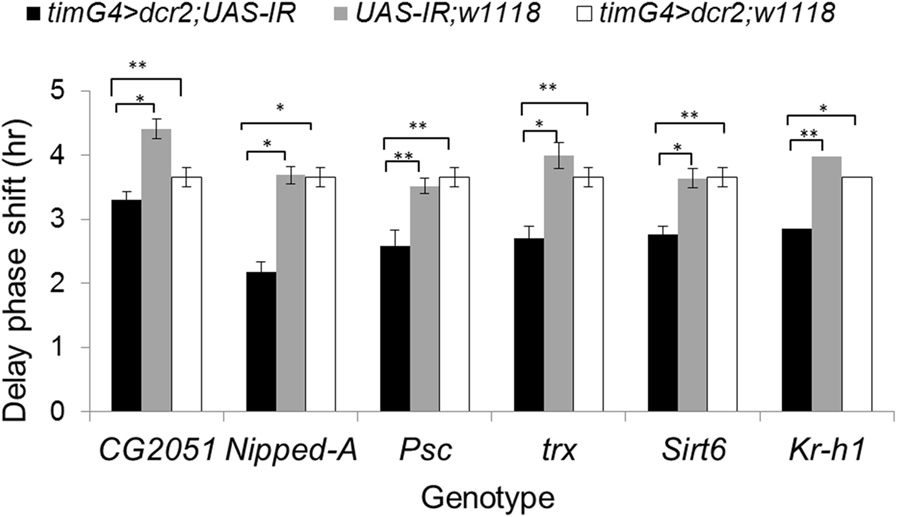
Phase response of flies with dsRNAi knockdown of chromatin remodelling genes. Phase response (delay) to ZT 15 light pulse, measured as the phase difference between pulsed and unpulsed flies. dsRNAi in clock neurons was driven in clock cells using a timGal4 driver (flies hemizygous for the two transgenes; black bars). In the control experiment, flies carried only the single UAS (grey) or the timGal4 (white) transgene. Note that the same set of Gal4 data is shown with each genotype for clarity. The plotted error bars signify the standard error mean for each genotype, and 32 flies were used per genotype. The asterisk indicates a significant difference between the treatment and the control (UAS and Gal4). The double asterisks (**) indicates when *p* < .0001, and the single asterisk (*) indicates when *p* < .05 from ANOVA with post hoc analysis using the Tukey test

Eight genes associated with chromatin remodelling were tested for their roles in circadian behaviour using available null mutants and dsRNAi transgenic flies (Table 1). Each gene was downregulated in clock neurons (and in photoreceptor cells in the compound eye) using the *tim-Gal4* driver with *UASdicer2*. The flies’ phase responses at ZT 15 were studied. The control flies expressed a single copy of either *UAS-IR* or *timG4 > dcr2* in the same genetic background as in the experimental line. There was no significant difference between the control and experimental flies in their light response, with a typical delay phase shift of 3.5–4 h (Fig. 2). Interestingly, the knockdown flies showed a significant reduction in their delay phase shift of about 1–2 h, as shown in Fig. 2.

**Fig. 3:**
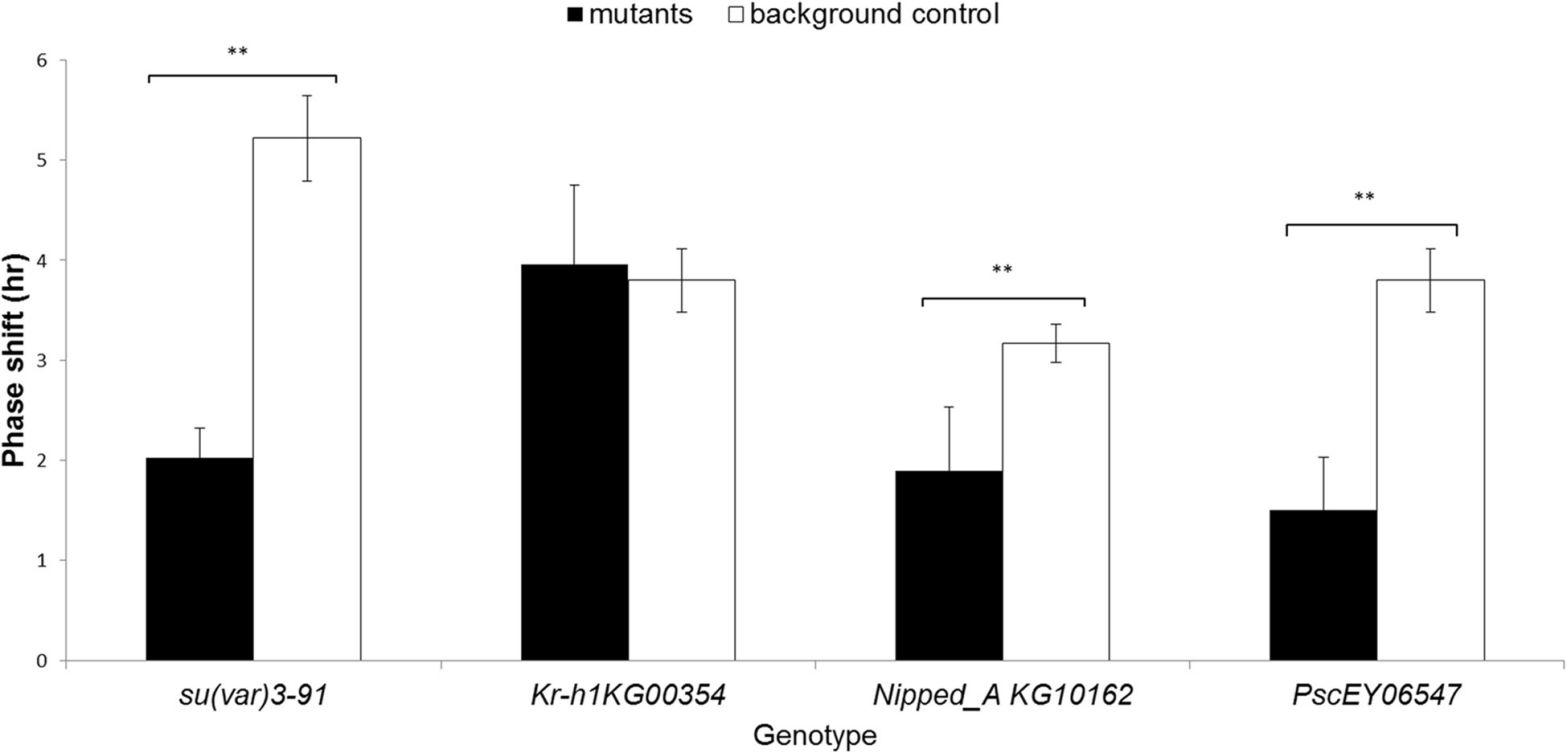
Light response in mutant flies and their background controls. Phase delays in locomotor activity following a light pulse at ZT 15 (relative to unpulsed flies) are shown. The error bars were based on SEM of each genotype from an independent *t* test (*n* = 32 flies). ** *p* < .0001; * *p* < .05

**Fig. 4:**
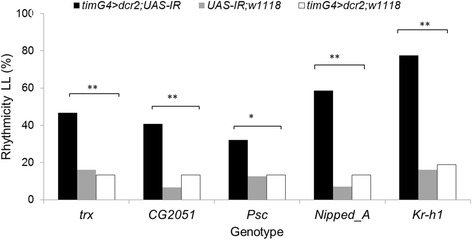
Knockdown of chromatin-related differentially expressed genes induces enhanced rhythmicity in constant light (LL). The proportion of flies (*n* = 32) that are rhythmic is shown. The error bars represent the SEM of each genotype, with 32 flies used per genotype. The asterisks indicate a significant difference between the treatment and the controls (UAS and Gal4, respectively). The double asterisk (**) indicates *p* < .0001 from ANOVA with post hoc analysis using the Tukey test. *p < .05, ** *p* < .01

We were unable to test the light response in *nejire* and *Su(var)3-9* RNAi knockdown flies because of lethality. However, we tested the light response of *Su(var)3-9* null mutants (Fig. 3). All the mutant strains were significantly less sensitive to the light pulse compared to their respective controls, with the exception of *Kr-h1*^KG00354^, which showed no significant difference from controls (Fig. 4). It is noteworthy that most of *Nipped*-A^KG10162^ and *Psc*^*EY06547*^ flies displayed a significantly reduced or complete lack of locomotor activity during the dark phase but became active again in the light phase (Additional file 1: Figure S5), representing prolonged uninterrupted bouts of sleep.

We also tested the effect of exposure to continuous light (LL) on the knockdown flies. The LL condition drives wild-type flies into arrhythmicity. This phenotype allows identification of mutants with defective circadian light input [14]. Under LL conditions, all the tested RNAi transgenic flies showed a significant increase in their rhythmicity compared to their respective controls, particularly with *Kr-h1* (χ ^2^ = 21.0, *p* < .001) and *Nipped-A* (χ ^2^ = 15.3, *p* < .001), where >50 % of flies were rhythmic (Fig. 4).

### Analysis of genes associated with ion channel and cellular communication

**Fig. 5:**
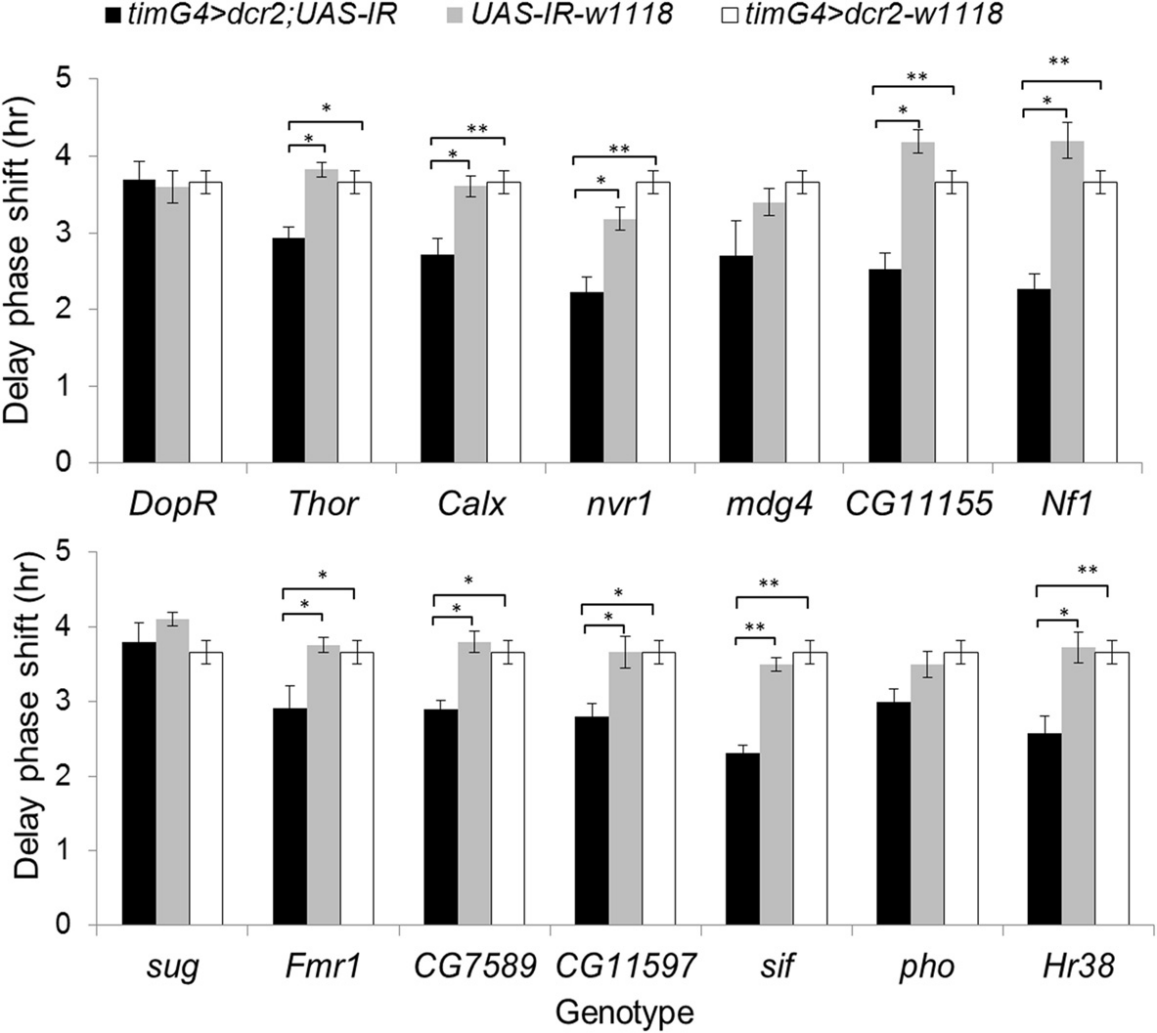
Light response of flies expressing dsRNAi targeting various differentially expressed genes. Phase delays in locomotor activity following a light pulse at ZT 15 (relative to unpulsed flies) are shown. The error bars were based on SEM of each genotype from an independent *t* test (*n* = 32 flies). ** *p* < .0001, * *p* < .05

We selected an additional 14 genes (Table 1), which represented various enriched functional categories in our DEGs. These categories included six genes for alternative splicing (downregulated) processes and eight genes for light response, neuronal communication, and sodium/calcium ionic balance processes. We tested the contribution of these genes to circadian behaviour using various dsRNAi knockdown transgenic flies. In response to a light stimulus at ZT 15, flies carrying a single transgene (*UAS-IR*; w1118 or *timG4 > dcr2; w1118*) responded with a typical delay of 3.5–4 h (Fig. 5). However, the expression of the RNAi resulted in a significantly reduced light response (approximately 1–2 h) in 10 out of the 14 tested genes (but not in *DopR*, *mdg4*, *sug* and *pho*).

**Fig. 6:**
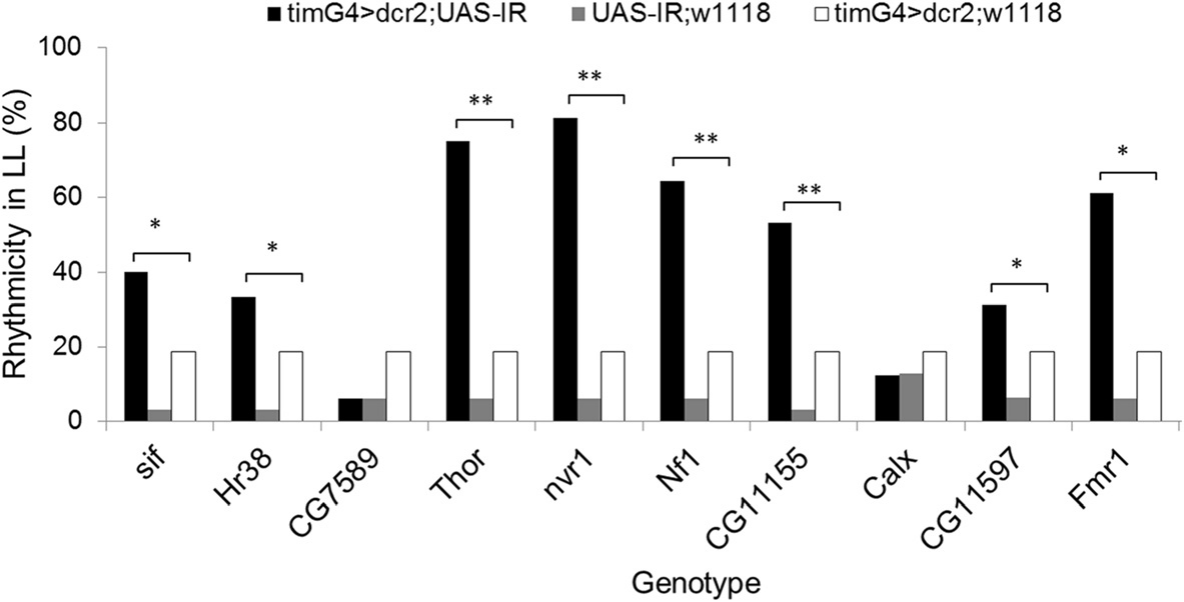
Behavioural rhythmicity in constant light (LL) in knockdown genotypes. Proportion of rhythmicity (asterisks and N, as in Fig. 4). Genotype, error bars, and significant p level are denoted as in Fig. 4

We also tested the activity of flies under LL (Fig. 6). Strikingly, the proportion of rhythmic individuals was substantially elevated in RNAi transgenic flies targeting *Thor* (proportion test, χ^2^ = 28.6 *p* < .001), *nrv1* (χ^2^ = 33.6, *p* < .001), *Nf1* (χ^2^ = 21.0, *p* < .001), *CG11155* (χ^2^ = 17.4, *p* < .001), and *Fmr1* (χ^2^ = 19.0, *p* < .001)*,* with 30 % to 80 % rhythmic flies.

## Discussion

Our aim was to identify genes whose expression is modulated by a light pulse during early night and explore their functional significance. We focused on the transcriptional response to early night stimulus (delay phase shift), as it generally induces a more robust response than the advanced phase shift in the late night [28]. We identified 200 genes for which transcript levels were significantly altered in response to a light pulse, at a false discovery rate cut-off of 10 % (Fig. 1).

Informed by our GO enrichment analysis, we characterised the roles of some genes associated with chromatin remodelling, ion channel activity, and cellular communication. Previous studies, mostly in mice, have revealed the role of histone methylation, acetylation, and deacetylation in the circadian clock [30, 31]. Light stimulation modulates histone remodelling in the mouse suprachiasmatic nucleus [32] by inducing phosphorylation of Ser-10 in H3 and acetylation at the promoters of *mPer1* or *mPer2* [33]. We noted that light pulses downregulate the expression of *nej* and *trithorax* in the fly brain (Table 1)*.* Both genes are known to drive histone acetylation and H3K4 methylation, a remodelling linked with gene activation. Another gene of interest, *Su(var)3-9,* is upregulated in response to light and is known to be associated with gene suppression [34]. NEJ was previously shown to act as a transcription co-activator via its histone acetyltransferase (HAT) activity [35, 36], which physically interacts with CLK and CLK-CYC through two of its binding sites [35]. If NEJ binding is required for activation of E-box containing clock genes, then its downregulation by a light pulse as evident from our data would result in attenuation of the circadian cycle and a delay. Trithorax, which was also downregulated in our data, interacts with NEJ to prevent Polycomb-mediated gene silencing by inhibiting H3Lys 27 trimethylation [37]. Indeed, our circadian behaviour results confirmed that changes in the expression of *trithorax* and *Su(var)3-9* affect circadian light sensitivity and the phase of rhythmic activities. Our data also suggest that histone remodelling, as in mammals, is involved in the dipteran clock light response, possibly by interacting with the CLK-CYC complex.

Another theme that was apparent among the enriched functions of light-induced transcripts related to cellular communication (ion channel transport, synaptic organisation, intracellular signalling cascade). For instance, the predicted molecular function of *CG11155* is ionotropic glutamate receptor activity, which mediates excitatory synaptic transmission. Glutamate has been previously shown to be essential for circadian light responses in mammals [22, 38], so perhaps downregulating *CG11155* by light stimulation reduced the clock’s light sensitivity by influencing glutamate activity in the fly. *Nervana1* (*nrv1*), another gene in this GO term, plays a critical role in regulating intracellular Ca^2+^ levels via the Na^+^/Ca^2+^ exchange mechanism [39]. By downregulating *nrv1* in the clock neurons, the flies’ responses to light were significantly reduced, and about 80 % of them maintained rhythmic activity in the LL condition. Interestingly, the other subunit of *nrv2* was also downregulated in response to light in this study, though it was not selected for further analysis. This again highlights a role for intracellular signalling and ion exchange in the clock’s light response. Interestingly, a recent imaging study of whole-brain explant cultures [40] reveals that a light pulse causes rapid desynchrony among clock cells, followed by gradual emergence of synchrony (‘phase retuning’). These findings underscore the importance of cellular communication genes in the light response.

The roles of the genes that we identified, such as *Nf1*, *nrv1*, and *CalX* in Ca^2+^ regulation, underscore the role of this pathway in the light response. This role was previously demonstrated in light-induced phase shifts in vertebrates [41]. The substantial rhythmicity in LL of *nrv1* and *Nf1 RNAi* flies (81 % and 64 %, respectively) suggests that these are important loci for circadian light input. In general, these findings reinforce the Njus-Sulzman-Hastings membrane model of the circadian clock [42], in which feedback interactions between membrane ion transport systems and ion concentration gradients modulate cell excitability and drive circadian oscillations.

Because we have profiled fly heads, our survey largely represents transcriptional changes in the compound eyes, given their large proportion of the volume of the fly’s head. This approach allows our data to be compared with the early circadian-clock studies, which also profiled fly heads [43–47]. However, many light-responsive DEGs may be expressed in non-clock cells. Additionally, we used the timGal4 driver in our functional assays. This driver is expressed both in clock neurons in the brain and in photoreceptors in the eyes [48]. Consequently, the effects that we see are mediated either via the eyes, via clock cells, or via both. However, three DEGs (*per*, *Kr-h1*, and *Thor)* are known to be enriched in clock cells [49, 50]. Furthermore, the lack of behavioural effect of genes that change in expression on early-light response might be due to their expression in non-TIM cells. Future experiments using techniques for profiling specific clock neurons [49, 51] would be valuable for identifying light input pathways within the clock.

## Conclusions

Our results show that the early transcriptional response to a pulse of light early at night involves a broad range of biological functions. In particular, this study invokes the role of intracellular cation balance and the role of chromatin remodelling in regulating light-induced phase changes in circadian behaviour.

## Methods

### *Drosophila* strains

All crosses were carried out at 25 °C on sugar medium (46.3 g sucrose; 46.3 g deactivated dry yeast; 10 g agar in 1 L of water; and 20 % Nipagin). The mutant lines were obtained from the Bloomington *Drosophila* Stock Centre at Indiana University, USA (Additional file 1: Table S4). All the dsRNAi stocks were obtained from the Vienna *Drosophila* RNAi Centre, Austria. The *Canton -S* (lab stock) was used as the wild-type. For all the RNAi crosses, the *tim- gal4* or *tim-gal4-UAS-Dicer-2 (timG4 > dcr2)* was used to drive the *UAS-IR* constructs [52]. RNAi experimental controls were generated by crossing the reporter (the *UAS-IR)* and driver (*timG4 > dcr2*) to *w*^*1118*^ flies.

### Behavioural analysis

We used the DAM2 *Drosophila* activity monitors (Trikinetics Inc., Waltham, MA, USA). In each experiment, 32 flies were used per genotype.

#### Phase response

Flies were entrained at 25 °C in LD (12:12) for 4 days and were allowed to free-run for 3 days in DD. The flies were then monitored for another week using the same entrainment regime (4 d in LD, 3 d in DD), but with a 30 min 1500 lux light pulse at ZT 15 on the last dark phase of the LD cycle. The time of activity offset in the second day in DD was used as a reference point for phase measurements. The phase of the first week (no pulse) was used as a reference phase, and the phase of the second week (light pulse) was used as a response phase. The phase shift was calculated as the difference between the reference phase and the response phase, with a negative value representing a delay phase shift (−∆Φ).

#### Locomotor activity

For measuring the circadian period in constant light (LL), the flies were entrained in LD 12:12 cycle for 4 days, and then were allowed to free-run for 7 days in at 25 °C. The free-run data was analysed with autocorrelation and spectral analysis using the CLEAN algorithm [53]. The activity of a fly was considered rhythmic when it showed a significant autocorrelation. A single detectable peak above the 99 % confidence limit in the spectral CLEAN analysis [53] was taken as the endogenous period. Individuals with multiple peaks above the 99 % confidence limit (CL) were considered to be displaying multiple rhythms, and any pattern below 99 % CL was regarded as arrhythmic.

### Sample collection and preparation

Young (aged 1–3 days) male *Canton-S* flies were entrained as described previously. The flies were divided into experimental and control groups. The experimental group received a 30-min light pulse at ZT 15, and the control group was left in constant darkness with no light stimulation. Flies were collected an hour after light stimulation under dark conditions (ZT 16.5), and snap frozen in liquid nitrogen. Each group was divided into four biological replicates, each containing about 1,000 flies. Total RNA was extracted from the heads using Triol (Invitrogen) according to the manufacturer’s instructions and purified with the RNeasy® MinElute™ Cleanup kit (Qiagen). RNA concentration was determined by using NanoDrop 2000 (Thermo Scientific), and sample integrity was assessed on an Agilent 2100 Bioanalyzer (Agilent Technologies, Santa Clara, CA, USA).

### Probe preparation, hybridisation, and processing

An Affymetrix GeneChip one-cycle target labelling kit (Affymetrix, Santa Clara, CA, USA) was used to generate cRNA from 5 μg total RNA according to the manufacturer’s protocol. The resulting biotin-labelled cRNA was fragmented and hybridised to the GeneChip *Drosophila* Genome Array 2 (Affymetrix). The post-hybridisation washing, staining, and detection using streptavidin-coupled fluorescent dye were done in the GeneChip Fluidics Station 400. The hybridised arrays were scanned using an Affymetrix GeneChip® 3000 scanner. Image generation and features extraction were performed using Affymetrix GeneChip® Operating Software and saved as cell intensity files. The experiment was performed at the University of Leicester Genome core facility, Leicester, UK.

### Microarray data processing

The scanned image (*.CEL) files were processed using the GeneChip Robust Multi-array Average method to adjust background, normalise datasets, and convert multiple probe values into a single expression value for each probe-set (gene). This was done using the R package (http://www.r-project.org/) and the Graphical User Interface of the *limma* package (affylmGUI) [54].

### Microarray data analysis

Quality control procedures included visual inspection of the chip pseudoimages and inspection of the histograms of raw signal intensity. The normalised data were analysed using the RankProd package [55] on an open-resource BioConductor project (http://bioconductor.org/) using a two-class model. RankProd is a non-parametric statistic that ranks all probe sets within each replicate by their expression level and then calculates each probe RankProd value, which depends on the number of times a particular probe set appears at the top (upregulated) or the bottom (downregulated) of the ranked list. The percent false positive value was calculated as an estimate of the false discovery rate for each probe set [55, 56]. The cut-off for significance genes was set at a false discovery rate threshold of 10 %.

### Validation of microarray data by qPCR

Total RNA was extracted from 250 fly heads, collected as previously described. Five biological replicates were used for each condition. The RNA samples were treated with 1 μl rDNase I (2 units/μL). cDNA was synthesised from 1 μg of the purified total RNA using 300 ng random primers (Promega) and the Stratagene AffinityScript™ Multiple Temperature Reverse kit in 20 μl reaction. The reactions were spiked with *aequorin* mRNA (0.5 μl/ 20 rxn) from jellyfish as an exogenous reference. The cDNA samples were diluted (4x), and 5 μl was used in subsequent reactions. We used the LightCycler® 480 Real-Time PCR System (Roche Applied Science). A standard curve was plotted for each of the genes, and five biological replicates were analysed for each condition. The cycle point was calculated by LightCycler software version 1.2 (Roche Diagnostics, GmbH, Germany) using the second derivative maximum method.

### Gene ontology functional enrichment for differentially expressed genes

All DEGs were grouped into GO categories of cellular component, biological process, and molecular function using clusterProfiler [29] in R. A hypergeometric test was used to establish a significant level (*p* < .05) for GO terms enrichment of the DEGs, and the *p* values were corrected for multiple testing.

### Statistical analysis

Parametric and non-parametric statistical tests were used according to whether the distribution of the data was normal and according to homogeneity of variance. Tests for normality were performed using Kolmogorov-Smirnov and Shapiro-Wilk tests. Levene’s test was used to assess homogeneity of variance. All statistical analyses were performed using the Statistical Package for the Social Sciences (SPSS), version 16.0.

### Availability of supporting data

The microarray data were deposited with the public GEO [57] databases under Accession number GSE39578. Other supporting data are included as Additional file 1.

## Additional file

Additional file 1: Table S1. List of differentially expressed genes (DEGs). **Table S2**. Validation of differentially expressed genes identified via microarray analysis by qPCR. **Table S3**. Gene Ontology (GO) categories enrichment. **Table S4**. Fly stocks. **Table S5**. qPCR primers information. **Figure S1**. Pairwise correlation among sample replicates. **Figure S2**. Enriched biological processes (BP) among DEG as reported by the clusterProfiler gene ontology analysis. The adjusted *P* value is color-coded as indicated in the key at the top. **Figure S3**. Enriched molecular functions (MF) among DEG as reported by the clusterProfiler gene ontology analysis. The adjusted *P* value is color-coded as indicated in the key at the top. **Figure S4**. Enriched cellular components (CC) among DEG as reported by the clusterProfiler gene ontology analysis. **Figure S5**. Activity profiles of Nipped-A KG10162 and PscEY06547 mutants. (PDF 665 kb)

## Abbreviations

FDR: False discovery rate
RNAi: RNA interference
DD: Continuous darkness
LL: Continuous light
DEGs: Differentially expressed genes
ZT: Zeitgeber time
UAS-IR: Upstream activated sequence fused to inverted repeat
GO: Gene ontology
