# Supplementary material for: Identification and functional analysis of early gene expression induced by circadian light-resetting in Drosophila
Source: BMC Genomics. 2015 Aug 1;16(1):570. doi: 10.1186/s12864-015-1787-7 (PMC4521455; doi:10.1186/s12864-015-1787-7)

Additional File 1 (Adewoye *et al*. 2015: Identification and functional analysis of early gene expression induced by circadian light-resetting in *Drosophila*)

Table S1. List of differentially expressed genes (DEG)

Flybase ID	Gene name	Fold change(log)	FDR
**FBgn0085708**	*CG41582*	-0.56	0.02
**FBgn0068673**	*Dsim\GD17454*	0.44	0.07
**FBgn0000017**	*abl kinase*	0.37	0.10
**FBgn0000038**	*AChR protein of Drosophila*	-0.29	0.09
**FBgn0011300**	*Activin A receptor 45A*	-0.46	0.06
**FBgn0041581**	*attacin*	0.65	0.03
**FBgn0012042**	*attacin*	0.59	0.06
**FBgn0034366**	*Autophagy-specific gene 7*	-0.39	0.10
**FBgn0010300**	*Brain Tumor*	-0.46	0.04
**FBgn0020908**	*calcium binding protein*	1.07	0.01
**FBgn0265784**	*cAMP-regulated enhancer-binding protein*	0.49	0.02
**FBgn0000279**	*Cecropin*	1.6	0.01
**FBgn0000278**	*Cecropin B*	1.28	0.00
**FBgn0038033**	*CG10097*	1.28	0.04
**FBgn0032707**	*CG10348*	-0.3	0.11
**FBgn0039311**	*CG10513*	1.42	0.01
**FBgn0039312**	*CG10514*	0.47	0.07
**FBgn0039927**	*CG11155*	-0.37	0.11
**FBgn0038733**	*CG11407*	0.49	0.02
**FBgn0029713**	*CG11436*	-0.35	0.11
**FBgn0039666**	*CG11501*	0.96	0.01
**FBgn0029584**	*CG11509*	-0.52	0.03
**FBgn0036249**	*CG11560*	0.34	0.09
**FBgn0039308**	*CG11889*	0.49	0.03
**FBgn0033558**	*CG12344*	-0.37	0.09
**FBgn0263997**	*CG12641*	0.46	0.07
**FBgn0032614**	*CG13284*	0.54	0.02
**FBgn0033784**	*CG13322*	0.46	0.04
**FBgn0034515**	*CG13428*	1.18	0.02
**FBgn0039255**	*CG13646*	-0.43	0.09
**FBgn0031939**	*CG13796*	0.68	0.02
**FBgn0085405**	*CG13845*	0.48	0.04
**FBgn0038532**	*CG14322*	0.55	0.01
**FBgn0033108**	*CG15236*	-0.42	0.07
**FBgn0030001**	*CG15335*	-0.61	0.00
**FBgn0031377**	*CG15356*	0.5	0.02
**FBgn0029801**	*CG15771*	0.46	0.06
**FBgn0260486**	*CG15819*	-0.43	0.04
**FBgn0031116**	*CG1695*	0.67	0.01

**FBgn0035142**	*CG17090*	-0.4	0.08
**FBgn0260941**	*CG17144*	-0.37	0.09
**FBgn0033936**	*CG17386*	-0.49	0.02
**FBgn0039959**	*CG17514*	-0.4	0.07
**FBgn0039856**	*CG1774*	0.41	0.09
**FBgn0263111**	*CG17816*	-0.49	0.03
**FBgn0039189**	*CG18528*	0.53	0.01
**FBgn0036091**	*CG18628*	2.23	0.05
**FBgn0265597**	*CG18646*	-0.54	0.02
**FBgn0040629**	*CG18673*	0.46	0.10
**FBgn0037469**	*CG1939*	0.34	0.11
**FBgn0037376**	*CG2051*	0.44	0.06
**FBgn0039902**	*CG2177*	-0.54	0.01
**FBgn0030240**	*CG2202*	0.36	0.08
**FBgn0030323**	*CG2371*	-0.38	0.06
**FBgn0029728**	*CG2861*	-0.43	0.03
**FBgn0050343**	*CG30343*	-0.41	0.06
**FBgn0261931**	*CG3099*	0.46	0.07
**FBgn0051326**	*CG31326*	0.39	0.01
**FBgn0034964**	*CG3173*	-0.5	0.02
**FBgn0264815**	*CG31757*	-0.4	0.04
**FBgn0263256**	*CG31900*	0.42	0.08
**FBgn0085432**	*CG32005*	-0.57	0.03
**FBgn0261555**	*CG32048*	0.38	0.09
**FBgn0263607**	*CG32155*	0.52	0.07
**FBgn0052185**	*CG32185*	1	0.01
**FBgn0052333**	*CG32333*	-0.42	0.09
**FBgn0052372**	*CG32372*	-0.37	0.09
**FBgn0052506**	*CG32506*	0.67	0.01
**FBgn0260741**	*CG3281*	0.42	0.04
**FBgn0052822**	*CG32822*	-0.54	0.02
**FBgn0032120**	*CG33298*	-0.37	0.08
**FBgn0085294**	*CG34265*	-0.34	0.09
**FBgn0034804**	*CG3832*	0.53	0.02
**FBgn0262124**	*CG40084*	-0.77	0.01
**FBgn0085553**	*CG41012*	-1.4	0.00
**FBgn0085553**	*CG41012*	-0.56	0.02
**FBgn0036629**	*CG4573*	-0.43	0.06
**FBgn0033815**	*CG4676*	0.6	0.01
**FBgn0259229**	*CG5156*	0.42	0.09
**FBgn0038934**	*CG5732*	-0.37	0.11
**FBgn0034717**	*CG5819*	0.4	0.07
**FBgn0032587**	*CG5953*	-0.44	0.04
**FBgn0029831**	*CG5966*	0.52	0.07
**FBgn0032264**	*CG6113*	0.38	0.07
**FBgn0030640**	*CG6294*	0.56	0.02

**FBgn0033872**	*CG6329*	0.38	0.08
**FBgn0259164**	*CG6600*	0.61	0.00
**FBgn0024941**	*CG6639*	-0.96	0.01
**FBgn0036969**	*CG6663*	1.98	0.02
**FBgn0035902**	*CG6683*	-0.4	0.05
**FBgn0037921**	*CG6808*	-0.68	0.00
**FBgn0037958**	*CG6962*	-0.35	0.07
**FBgn0025687**	*CG7144*	-0.46	0.02
**FBgn0036727**	*CG7589*	0.41	0.07
**FBgn0038631**	*CG7695*	0.48	0.07
**FBgn0039737**	*CG7920*	0.44	0.07
**FBgn0037610**	*CG8043*	0.45	0.11
**FBgn0033998**	*CG8092*	0.62	0.04
**FBgn0037684**	*CG8129*	0.64	0.00
**FBgn0034143**	*CG8303*	0.48	0.04
**FBgn0033730**	*CG8511*	-0.52	0.05
**FBgn0084933**	*CG9007*	-0.38	0.11
**FBgn0031832**	*CG9596*	-0.42	0.07
**FBgn0038211**	*CG9649*	0.45	0.04
**FBgn0029948**	*CheA7a*	0.46	0.08
**FBgn0000363**	*couch potato*	-0.39	0.08
**FBgn0261617**	*CREB binding protein*	-0.42	0.06
**FBgn0000317**	*Crinkled*	-0.39	0.08
**FBgn0038681**	*Cyp12a4*	-0.56	0.03
**FBgn0031432**	*Cyp309a1*	0.47	0.05
**FBgn0038006**	*Cyp313a2*	-0.44	0.07
**FBgn0030615**	*Cyp4s3*	-0.38	0.10
**FBgn0024913**	*dActivin*	-0.36	0.09
**FBgn0037212**	*Dalpha4*	-0.59	0.02
**FBgn0032151**	*Dalpha6*	-0.47	0.10
**FBgn0260866**	*Defense repressor 1*	-0.36	0.10
**FBgn0034407**	*diptericin-like protein*	0.73	0.04
**FBgn0086525**	*Dmir\CG3831*	0.53	0.02
**FBgn0028996**	*D-onecut*	-0.43	0.03
**FBgn0011582**	*dopamine D1 receptor*	-0.52	0.03
**FBgn0010388**	*Drosocin*	0.52	0.08
**FBgn0010381**	*Drosomycin*	0.46	0.10
**FBgn0000567**	*ecdysone inducible protein*	-0.4	0.08
**FBgn0263755**	*Enhancer of variegation 3-9*	0.48	0.02
**FBgn0024836**	*flamingo*	-0.55	0.02
**FBgn0033079**	*Flavin-containing monooxygenase 2*	0.41	0.08
**FBgn0038197**	*forkhead box, sub-group O*	-0.39	0.09
**FBgn0028734**	*Fragile X*	0.5	0.02
**FBgn0011592**	*frazzled*	-0.47	0.03
**FBgn0003997**	*head involution defect*	-0.38	0.07
**FBgn0001225**	*heat shock protein hsp26*	0.42	0.08

**FBgn0010303**	*hemipterous*	0.44	0.03
**FBgn0053864**	*His1:CG33864*	-1.06	0.00
**FBgn0061209**	*His2B:CG17949*	-0.64	0.02
**FBgn0053910**	*His2B:CG33910*	-0.64	0.02
**FBgn0014859**	*Hormone receptor-like in 38*	0.8	0.01
**FBgn0262735**	*IGF-II mRNA-binding protein*	-0.35	0.11
**FBgn0024963**	*indefinite*	-0.48	0.04
**FBgn0261560**	*insulin-stimulated eIF-4E binding protein*	-0.36	0.09
**FBgn0028420**	*Krueppel-homolog alpha-isoform*	-0.37	0.09
**FBgn0013576**	*late puff gene at 82F*	-0.51	0.02
**FBgn0040104**	*Lectin24A*	0.33	0.06
**FBgn0025726**	*lethal (4) ry16*	-0.42	0.10
**FBgn0030993**	*Mec2*	0.36	0.04
**FBgn0003460**	*medusa*	-0.38	0.09
**FBgn0020270**	*meiotic recombination 11*	-0.31	0.11
**FBgn0028416**	*Met75Ca*	4.31	0.01
**FBgn0028415**	*Met75Cb*	4.31	0.01
**FBgn0019985**	*metabotropic glutamate receptor*	-0.47	0.08
**FBgn0014865**	*Metchnikowin*	0.58	0.05
**FBgn0265487**	*mindmelt*	-0.71	0.00
**FBgn0002781**	*Modifier67.2*	-0.42	0.04
**FBgn0040299**	*myosin VII 28B1-B4*	-0.49	0.03
**FBgn0013995**	*Na/Ca-exchange protein*	-0.38	0.08
**FBgn0015609**	*N-cadherin*	-0.36	0.09
**FBgn0015776**	*Nervana*	-0.34	0.11
**FBgn0015777**	*nervana2*	-0.38	0.08
**FBgn0015269**	*neurofibromatosis type 1*	-0.51	0.02
**FBgn0263111**	*nightblind*	-0.52	0.02
**FBgn0053554**	*Nipped*	-0.46	0.03
**FBgn0043530**	*Odorant-binding protein 51a*	1.46	0.11
**FBgn0034470**	*Odorant-binding protein 56d*	-0.49	0.04
**FBgn0030103**	*Odorant-binding protein 8a*	0.42	0.11
**FBgn0039678**	*Odorant-binding protein 99a*	-0.46	0.00
**FBgn0010403**	*Olfactory-specific E*	-0.73	0.00
**FBgn0037906**	*Peptidoglycan recognition protein LB*	0.74	0.00
**FBgn0003068**	*period clock protein*	-0.5	0.02
**FBgn0043575**	*PGRP-SC2*	1.14	0.00
**FBgn0011293**	*Pherokine 1*	-0.41	0.06
**FBgn0011279**	*Pheromone-binding protein-related protein 1*	-0.83	0.00
**FBgn0259225**	*Phosphodiesterase 1c*	-0.53	0.02
**FBgn0003416**	*phospholipase Cgamma*	-0.37	0.08
**FBgn0011754**	*phosphorylase kinase gamma*	0.51	0.06
**FBgn0002521**	*Pleiohomeotic*	-0.39	0.08
**FBgn0025741**	*Plexin A*	-0.44	0.04

**FBgn0005624**	*Posterior sex combs*	0.48	0.04
**FBgn0022724**	*prodos*	-0.34	0.10
**FBgn0004181**	*Protein ejaculatory bulb*	3.09	0.00
**FBgn0011694**	*Protein ejaculatory bulb II*	3.28	0.00
**FBgn0085434**	*put. sodium channel gene*	-0.45	0.04
**FBgn0032638**	*Regulator of G-protein signalling 7*	-0.96	0.01
**FBgn0039883**	*RhoGAP100F*	-0.6	0.01
**FBgn0036518**	*RhoGAP71E*	-0.48	0.03
**FBgn0265598**	*rhombotin*	-0.37	0.09
**FBgn0011705**	*rolling stone*	0.43	0.07
**FBgn0011286**	*ryanodine receptor*	-0.46	0.04
**FBgn0032726**	*selenocysteine methyltransferase*	0.55	0.03
**FBgn0003380**	*Shaker*	-1	0.00
**FBgn0003380**	*Shaker*	-0.59	0.02
**FBgn0032840**	*short neuropeptide F-1*	-0.38	0.09
**FBgn0037802**	*Sirt6*	0.36	0.08
**FBgn0029761**	*small conductance calcium-activated potassium channel*	-0.44	0.04
**FBgn0085450**	*Sno-oncogene*	-0.38	0.09
**FBgn0002609**	*split locus enhancer protein m3*	-0.37	0.10
**FBgn0024189**	*sticks and stones*	-0.45	0.06
**FBgn0085447**	*still life type 1*	-0.58	0.02
**FBgn0033782**	*sugarbabe*	-0.82	0.00
**FBgn0262614**	*tamou*	-0.36	0.07
**FBgn0024921**	*transportin*	-0.48	0.04
**FBgn0003862**	*trithorax*	-0.41	0.06
**FBgn0003721**	*Tropomyosin*	0.37	0.10
**FBgn0004169**	*troponin T*	-0.65	0.00
**FBgn0044812**	*Turandot C*	0.45	0.03
**FBgn0031701**	*Turandot M*	1.19	0.01
**FBgn0010473**	*turtle*	-0.46	0.02
**FBgn0026314**	*UDP-glycosyltransferase*	0.34	0.10
**FBgn0034013**	*unc-5-like*	-0.38	0.08
**FBgn0004395**	*unkempt*	-0.42	0.07
**FBgn0027101**	*unknown-telomeric-protein-gene*	-0.38	0.11
**FBgn0030354**	*Upf1*	-0.44	0.06
**FBgn0003997**	*wrinkled*	-0.41	0.07
**FBgn0004047**	*yolk protein*	-1.23	0.00
**FBgn0005391**	*yolk protein*	-1.47	0.01
**FBgn0004045**	*yolk protein 1*	-1.63	0.00
**FBgn0061476**	*zwilch*	-0.44	0.06

Table S2. Validation of differentially expressed genes identified via microarray analysis by qPCR.

**Gene**	**Fold change microarray**	**Fold change qPCR**	**p-value**	**Expression**
sug	-0.82	0.14	0.03	Up
Fmr1	0.50	0.49	0.06	Up
Hr38 *	0.80	0.63	0.03	Up
per *	-0.50	-0.52	0.03	Down
CG3099 *	0.46	0.24	0.03	Up
Psc	0.48	0.16	0.23	Up
CG2051 *	-0.44	-0.08	0.04	Down
CG11597	0.48	0.70	0.10	Up
Thor *	-0.36	-0.99	0.00	Down
Su(var) 3-9 *	0.48	0.43	0.04	Up
so *	-0.38	-0.71	0.01	Down
CG1379 *	-0.68	-0.18	0.03	Down
Kr-h1 *	-0.37	-0.45	0.04	Down
hep	0.44	-0.25	0.31	Down
Yp1	-1.63	-3.75	0.07	Down
Sirt6	0.36	-0.21	0.18	Down

Table S3. Gene Ontology (GO) categories enrichment (ClusterProfiler analysis)

Molecular function

ID	Description	pvalue	p.adjust
GO:0003674	molecular_function	9.42E-09	7.53E-07
GO:0022836	gated channel activity	7.56E-07	3.02E-05
GO:0005231	excitatory extracellular ligand-gated ion channel activity	4.71E-06	0.000125516
GO:0022892	substrate-specific transporter activity	9.62E-06	0.000172319
GO:0005230	extracellular ligand-gated ion channel activity	1.08E-05	0.000172319
GO:0015075	ion transmembrane transporter activity	1.41E-05	0.000187771
GO:0005216	ion channel activity	1.89E-05	0.000200976
GO:0005515	protein binding	2.01E-05	0.000200976
GO:0022838	substrate-specific channel activity	2.37E-05	0.000203216
GO:0022891	substrate-specific transmembrane transporter activity	2.54E-05	0.000203216
GO:0015267	channel activity	3.51E-05	0.000233831
GO:0022803	passive transmembrane transporter activity	3.51E-05	0.000233831
GO:0005488	binding	5.47E-05	0.000336556
GO:0005261	cation channel activity	6.81E-05	0.000389358
GO:0005099	Ras GTPase activator activity	8.49E-05	0.000452749
GO:0015276	ligand-gated ion channel activity	0.000126495	0.000579812
GO:0022834	ligand-gated channel activity	0.000126495	0.000579812
GO:0008324	cation transmembrane transporter activity	0.000130458	0.000579812
GO:0046872	metal ion binding	0.00014536	0.000612043
GO:0043167	ion binding	0.000191172	0.000749424
GO:0043169	cation binding	0.000196724	0.000749424
GO:0022857	transmembrane transporter activity	0.000223062	0.000811135
GO:0005215	transporter activity	0.000275081	0.000956804
GO:0046873	metal ion transmembrane transporter activity	0.000346909	0.001156363
GO:0005083	small GTPase regulator activity	0.000438074	0.001401838
GO:0005096	GTPase activator activity	0.000469214	0.001443735
GO:0030695	GTPase regulator activity	0.000938362	0.002780331
GO:0004871	signal transducer activity	0.001047747	0.002890337
GO:0060089	molecular transducer activity	0.001047747	0.002890337
GO:0060589	nucleoside-triphosphatase regulator activity	0.001313544	0.003502785
GO:0008270	zinc ion binding	0.001420997	0.003667089
GO:0046914	transition metal ion binding	0.002171633	0.005429082
GO:0008047	enzyme activator activity	0.002628504	0.006372132
GO:0003677	DNA binding	0.005049115	0.011880272
GO:0022890	inorganic cation transmembrane transporter activity	0.005700361	0.013029396
GO:0008134	transcription factor binding	0.006416071	0.014257935
GO:0038023	signaling receptor activity	0.01030239	0.022275438
GO:0004872	receptor activity	0.014454308	0.030430122

GO:0043565	sequence-specific DNA binding	0.014986562	0.030741665
GO:0001071	nucleic acid binding transcription factor activity	0.024209814	0.047238661
GO:0003700	sequence-specific DNA binding transcription factor activity	0.024209814	0.047238661

Biological processes

ID	Description	pvalue	p.adjust
GO:0009605	response to external stimulus	6.81E-16	2.84E-13
GO:0050896	response to stimulus	2.59E-13	5.41E-11
GO:0050830	defense response to Gram-positive bacterium	1.60E-12	2.23E-10
GO:0009617	response to bacterium	8.29E-12	8.64E-10
GO:0065007	biological regulation	1.49E-11	1.24E-09
GO:0050789	regulation of biological process	3.88E-11	2.70E-09
GO:0050794	regulation of cellular process	6.65E-11	3.96E-09
GO:0051239	regulation of multicellular organismal process	2.52E-10	1.31E-08
GO:0019731	antibacterial humoral response	3.12E-10	1.37E-08
GO:0048583	regulation of response to stimulus	3.28E-10	1.37E-08
GO:0042221	response to chemical	6.23E-10	2.36E-08
GO:0002376	immune system process	1.09E-09	3.32E-08
GO:0043207	response to external biotic stimulus	1.13E-09	3.32E-08
GO:0051707	response to other organism	1.13E-09	3.32E-08
GO:0009607	response to biotic stimulus	1.27E-09	3.32E-08
GO:0042742	defense response to bacterium	1.28E-09	3.32E-08
GO:0030030	cell projection organization	3.48E-09	8.12E-08
GO:0044699	single-organism process	3.50E-09	8.12E-08
GO:0098542	defense response to other organism	3.97E-09	8.72E-08
GO:0031175	neuron projection development	4.91E-09	9.96E-08
GO:0032501	multicellular organismal process	5.02E-09	9.96E-08
GO:0044707	single-multicellular organism process	6.52E-09	1.24E-07
GO:0030182	neuron differentiation	6.94E-09	1.26E-07
GO:0048812	neuron projection morphogenesis	1.50E-08	2.60E-07
GO:0009653	anatomical structure morphogenesis	2.57E-08	4.29E-07
GO:0048699	generation of neurons	4.26E-08	6.72E-07
GO:0019222	regulation of metabolic process	4.35E-08	6.72E-07
GO:0006952	defense response	5.07E-08	7.56E-07
GO:0051704	multi-organism process	5.49E-08	7.73E-07
GO:0007154	cell communication	5.63E-08	7.73E-07
GO:0048667	cell morphogenesis involved in neuron differentiation	5.86E-08	7.73E-07
GO:0048731	system development	6.02E-08	7.73E-07
GO:0048666	neuron development	6.12E-08	7.73E-07
GO:0000904	cell morphogenesis involved in differentiation	6.31E-08	7.74E-07
GO:0010646	regulation of cell communication	7.95E-08	9.47E-07
GO:0048858	cell projection morphogenesis	9.45E-08	1.08E-06
GO:0007623	circadian rhythm	9.55E-08	1.08E-06
GO:0032990	cell part morphogenesis	1.10E-07	1.21E-06

GO:0048511	rhythmic process	1.15E-07	1.23E-06
GO:0009887	organ morphogenesis	1.29E-07	1.35E-06
GO:0080090	regulation of primary metabolic process	2.17E-07	2.20E-06
GO:0023052	signaling	2.27E-07	2.20E-06
GO:0044700	single organism signaling	2.27E-07	2.20E-06
GO:0050829	defense response to Gram-negative bacterium	2.36E-07	2.23E-06
GO:0008150	biological_process	2.69E-07	2.45E-06
GO:0006955	immune response	2.71E-07	2.45E-06
GO:0022008	neurogenesis	3.16E-07	2.81E-06
GO:0031323	regulation of cellular metabolic process	3.39E-07	2.94E-06
GO:0006811	ion transport	4.73E-07	4.02E-06
GO:0006950	response to stress	6.41E-07	5.35E-06
GO:0006959	humoral immune response	6.93E-07	5.66E-06
GO:0007275	multicellular organismal development	7.28E-07	5.84E-06
GO:0023051	regulation of signaling	8.24E-07	6.48E-06
GO:0019730	antimicrobial humoral response	9.09E-07	7.02E-06
GO:0010975	regulation of neuron projection development	1.13E-06	8.58E-06
GO:0031344	regulation of cell projection organization	1.25E-06	9.30E-06
GO:0007409	axonogenesis	1.29E-06	9.43E-06
GO:0009628	response to abiotic stimulus	1.33E-06	9.53E-06
GO:0032502	developmental process	1.57E-06	1.10E-05
GO:0000902	cell morphogenesis	1.58E-06	1.10E-05
GO:0061564	axon development	1.79E-06	1.22E-05
GO:0030154	cell differentiation	2.03E-06	1.35E-05
GO:0007399	nervous system development	2.04E-06	1.35E-05
GO:0048513	organ development	2.35E-06	1.53E-05
GO:0050793	regulation of developmental process	2.42E-06	1.55E-05
GO:0007552	metamorphosis	3.09E-06	1.95E-05
GO:0003008	system process	3.26E-06	2.03E-05
GO:0031329	regulation of cellular catabolic process	3.45E-06	2.11E-05
GO:0040007	growth	3.54E-06	2.14E-05
GO:0050795	regulation of behavior	3.71E-06	2.21E-05
GO:0051128	regulation of cellular component organization	4.55E-06	2.67E-05
GO:0044767	single-organism developmental process	4.74E-06	2.74E-05
GO:0048707	instar larval or pupal morphogenesis	5.64E-06	3.22E-05
GO:0060255	regulation of macromolecule metabolic process	5.90E-06	3.32E-05
GO:0048512	circadian behavior	5.97E-06	3.32E-05
GO:0048869	cellular developmental process	6.38E-06	3.50E-05
GO:0016043	cellular component organization	6.62E-06	3.58E-05
GO:0007622	rhythmic behavior	7.16E-06	3.79E-05
GO:0032989	cellular component morphogenesis	7.17E-06	3.79E-05
GO:0009886	post-embryonic morphogenesis	7.54E-06	3.93E-05
GO:0009894	regulation of catabolic process	7.73E-06	3.98E-05
GO:0045475	locomotor rhythm	7.85E-06	3.99E-05
GO:0051171	regulation of nitrogen compound metabolic process	9.87E-06	4.96E-05

GO:0007411	axon guidance	1.01E-05	4.96E-05
GO:0048813	dendrite morphogenesis	1.01E-05	4.96E-05
GO:0016358	dendrite development	1.22E-05	5.91E-05
GO:0048522	positive regulation of cellular process	1.25E-05	6.02E-05
GO:0045595	regulation of cell differentiation	1.28E-05	6.08E-05
GO:0071840	cellular component organization or biogenesis	1.36E-05	6.29E-05
GO:0016049	cell growth	1.36E-05	6.29E-05
GO:0019219	regulation of nucleobase-containing compound metabolic process	1.37E-05	6.29E-05
GO:0097485	neuron projection guidance	1.41E-05	6.32E-05
GO:0065008	regulation of biological quality	1.42E-05	6.32E-05
GO:0042330	taxis	1.43E-05	6.32E-05
GO:0007610	behavior	1.46E-05	6.39E-05
GO:0008340	determination of adult lifespan	1.51E-05	6.58E-05
GO:0031326	regulation of cellular biosynthetic process	1.59E-05	6.83E-05
GO:0048518	positive regulation of biological process	1.62E-05	6.83E-05
GO:0009889	regulation of biosynthetic process	1.62E-05	6.83E-05
GO:0045935	positive regulation of nucleobase-containing compound metabolic process	1.73E-05	7.21E-05
GO:0048569	post-embryonic organ development	1.77E-05	7.33E-05
GO:0010259	multicellular organismal aging	1.79E-05	7.34E-05
GO:0031325	positive regulation of cellular metabolic process	1.83E-05	7.40E-05
GO:0007268	synaptic transmission	1.94E-05	7.77E-05
GO:0051960	regulation of nervous system development	2.08E-05	8.27E-05
GO:0007568	aging	2.12E-05	8.33E-05
GO:0009266	response to temperature stimulus	2.15E-05	8.37E-05
GO:0006935	chemotaxis	2.37E-05	9.15E-05
GO:0050803	regulation of synapse structure and activity	2.43E-05	9.31E-05
GO:0007267	cell-cell signaling	2.56E-05	9.69E-05
GO:0007560	imaginal disc morphogenesis	2.60E-05	9.70E-05
GO:0048563	post-embryonic organ morphogenesis	2.60E-05	9.70E-05
GO:0009893	positive regulation of metabolic process	2.67E-05	9.87E-05
GO:0007165	signal transduction	2.87E-05	0.000104476
GO:0048468	cell development	2.88E-05	0.000104476
GO:0044708	single-organism behavior	3.05E-05	0.000109782
GO:0080134	regulation of response to stress	3.13E-05	0.000111696
GO:0044763	single-organism cellular process	3.19E-05	0.000112907
GO:0010468	regulation of gene expression	3.37E-05	0.000118136
GO:0040008	regulation of growth	3.44E-05	0.000118682
GO:0030431	sleep	3.47E-05	0.000118682
GO:0048856	anatomical structure development	3.49E-05	0.000118682
GO:0051716	cellular response to stimulus	3.50E-05	0.000118682
GO:0040011	locomotion	3.74E-05	0.000125659
GO:2000026	regulation of multicellular organismal development	3.94E-05	0.000131477
GO:0045664	regulation of neuron differentiation	4.24E-05	0.000140387

GO:0051173	positive regulation of nitrogen compound metabolic process	4.28E-05	0.000140387
GO:0009791	post-embryonic development	4.39E-05	0.00014305
GO:0051254	positive regulation of RNA metabolic process	4.45E-05	0.00014388
GO:2000112	regulation of cellular macromolecule biosynthetic process	4.66E-05	0.000149474
GO:0010556	regulation of macromolecule biosynthetic process	4.74E-05	0.000150931
GO:0048749	compound eye development	4.81E-05	0.000152019
GO:0002682	regulation of immune system process	5.56E-05	0.000174436
GO:0007635	chemosensory behavior	5.80E-05	0.000180432
GO:0009966	regulation of signal transduction	5.88E-05	0.000181688
GO:0002165	instar larval or pupal development	5.93E-05	0.000181794
GO:0050767	regulation of neurogenesis	6.12E-05	0.000186257
GO:0035114	imaginal disc-derived appendage morphogenesis	7.27E-05	0.000219705
GO:0010033	response to organic substance	7.35E-05	0.00022052
GO:0035107	appendage morphogenesis	7.67E-05	0.000228475
GO:0048737	imaginal disc-derived appendage development	8.09E-05	0.000237866
GO:0007444	imaginal disc development	8.10E-05	0.000237866
GO:0048736	appendage development	8.53E-05	0.000248704
GO:0006357	regulation of transcription from RNA polymerase II promoter	8.99E-05	0.000260293
GO:0010769	regulation of cell morphogenesis involved in differentiation	9.49E-05	0.000272853
GO:0001654	eye development	9.72E-05	0.000277594
GO:0048584	positive regulation of response to stimulus	0.000117393	0.00033086
GO:0045893	positive regulation of transcription, DNA-templated	0.000117428	0.00033086
GO:0050808	synapse organization	0.000119125	0.000333389
GO:0042048	olfactory behavior	0.000120684	0.000335501
GO:0030001	metal ion transport	0.000124464	0.000343719
GO:0007423	sensory organ development	0.000146128	0.000400892
GO:0010628	positive regulation of gene expression	0.000153656	0.000418787
GO:0042127	regulation of cell proliferation	0.000157443	0.000426322
GO:1902680	positive regulation of RNA biosynthetic process	0.000158795	0.00042721
GO:0006928	cellular component movement	0.00016015	0.000428092
GO:0060284	regulation of cell development	0.00018668	0.000495831
GO:0044765	single-organism transport	0.000193524	0.000510756
GO:0007476	imaginal disc-derived wing morphogenesis	0.000196281	0.000514776
GO:0035120	post-embryonic appendage morphogenesis	0.000203877	0.000531356
GO:0050877	neurological system process	0.000227206	0.000588477
GO:0007472	wing disc morphogenesis	0.000230502	0.000593329
GO:0010604	positive regulation of macromolecule metabolic process	0.000242997	0.00062132
GO:0009891	positive regulation of biosynthetic process	0.000247244	0.00062132
GO:0031328	positive regulation of cellular biosynthetic process	0.000247244	0.00062132

GO:0001736	establishment of planar polarity	0.000248826	0.00062132
GO:0007164	establishment of tissue polarity	0.000248826	0.00062132
GO:0051252	regulation of RNA metabolic process	0.000257543	0.000639258
GO:0051179	localization	0.00027722	0.000684027
GO:0032101	regulation of response to external stimulus	0.000279815	0.000686369
GO:0007626	locomotory behavior	0.00029459	0.000718385
GO:0010557	positive regulation of macromolecule biosynthetic process	0.000315501	0.000764907
GO:0035220	wing disc development	0.000352749	0.000850267
GO:0009987	cellular process	0.000368542	0.000883229
GO:0008038	neuron recognition	0.000371014	0.000884074
GO:1902531	regulation of intracellular signal transduction	0.000376094	0.000891087
GO:0008037	cell recognition	0.000391826	0.000923116
GO:0031667	response to nutrient levels	0.000436252	0.001022006
GO:0042063	gliogenesis	0.000443945	0.001034217
GO:0001738	morphogenesis of a polarized epithelium	0.000484614	0.001116485
GO:0009991	response to extracellular stimulus	0.000484614	0.001116485
GO:0006355	regulation of transcription, DNA-templated	0.000498957	0.001136968
GO:2001141	regulation of RNA biosynthetic process	0.000498957	0.001136968
GO:0007618	mating	0.000506142	0.001147071
GO:0006812	cation transport	0.000521072	0.001174526
GO:0019098	reproductive behavior	0.000600573	0.001346447
GO:0006366	transcription from RNA polymerase II promoter	0.000650527	0.001450641
GO:0080135	regulation of cellular response to stress	0.000687855	0.001525722
GO:0051963	regulation of synapse assembly	0.0007298	0.001610193
GO:0022604	regulation of cell morphogenesis	0.000832166	0.001826385
GO:0048523	negative regulation of cellular process	0.000840259	0.001834492
GO:0001745	compound eye morphogenesis	0.000869659	0.001888791
GO:0042594	response to starvation	0.000970033	0.002089887
GO:0009719	response to endogenous stimulus	0.000972273	0.002089887
GO:0010647	positive regulation of cell communication	0.000999446	0.002137276
GO:0022603	regulation of anatomical structure morphogenesis	0.001029547	0.002190413
GO:0006351	transcription, DNA-templated	0.001113842	0.002357727
GO:0007617	mating behavior	0.001140641	0.002390637
GO:0007613	memory	0.001140855	0.002390637
GO:0032774	RNA biosynthetic process	0.001161019	0.002420724
GO:0050807	regulation of synapse organization	0.001403424	0.002911581
GO:0007600	sensory perception	0.001447049	0.00297251
GO:0048592	eye morphogenesis	0.001447049	0.00297251
GO:1901136	carbohydrate derivative catabolic process	0.001457057	0.002978397
GO:0031347	regulation of defense response	0.001475734	0.00300186
GO:1901700	response to oxygen-containing compound	0.001554156	0.003146034
GO:0007416	synapse assembly	0.001595572	0.003198815
GO:0007528	neuromuscular junction development	0.001595572	0.003198815
GO:0006810	transport	0.001644738	0.003281607
GO:0009416	response to light stimulus	0.001728791	0.003432886

GO:0051336	regulation of hydrolase activity	0.001798564	0.003554509
GO:0051093	negative regulation of developmental process	0.001982123	0.003887099
GO:0044712	single-organism catabolic process	0.001985497	0.003887099
GO:0006914	autophagy	0.002261403	0.004371462
GO:0048519	negative regulation of biological process	0.002262514	0.004371462
GO:0044706	multi-multicellular organism process	0.002264355	0.004371462
GO:0007611	learning or memory	0.002350277	0.004495714
GO:0050890	cognition	0.002350277	0.004495714
GO:0044705	multi-organism reproductive behavior	0.002623206	0.004972167
GO:0046434	organophosphate catabolic process	0.002623206	0.004972167
GO:0009888	tissue development	0.002791595	0.005267399
GO:0045944	positive regulation of transcription from RNA polymerase II promoter	0.0028151	0.005287823
GO:0065009	regulation of molecular function	0.003000898	0.005611544
GO:0051234	establishment of localization	0.003075266	0.005718434
GO:0070887	cellular response to chemical stimulus	0.003085486	0.005718434
GO:0051129	negative regulation of cellular component organization	0.003327674	0.006139999
GO:0034654	nucleobase-containing compound biosynthetic process	0.003380419	0.006209844
GO:0009890	negative regulation of biosynthetic process	0.003419521	0.006226813
GO:0031327	negative regulation of cellular biosynthetic process	0.003419521	0.006226813
GO:0044087	regulation of cellular component biogenesis	0.003473203	0.006269808
GO:0051705	multi-organism behavior	0.003473203	0.006269808
GO:0010608	posttranscriptional regulation of gene expression	0.003837348	0.006897303
GO:0006139	nucleobase-containing compound metabolic process	0.003874322	0.006933873
GO:0048589	developmental growth	0.004100884	0.007307985
GO:0045596	negative regulation of cell differentiation	0.004221873	0.00749158
GO:0006184	GTP catabolic process	0.004386626	0.007750945
GO:0016070	RNA metabolic process	0.004619779	0.008128471
GO:0044271	cellular nitrogen compound biosynthetic process	0.004685197	0.008208937
GO:1901069	guanosine-containing compound catabolic process	0.004730315	0.008238933
GO:1901565	organonitrogen compound catabolic process	0.004741832	0.008238933
GO:0090066	regulation of anatomical structure size	0.004806821	0.008307024
GO:0007389	pattern specification process	0.004820863	0.008307024
GO:0090304	nucleic acid metabolic process	0.004885753	0.008384193
GO:0046039	GTP metabolic process	0.005093443	0.008669248
GO:0048638	regulation of developmental growth	0.005093443	0.008669248
GO:0051246	regulation of protein metabolic process	0.005191105	0.008799556
GO:0009314	response to radiation	0.005274604	0.008811202
GO:0009146	purine nucleoside triphosphate catabolic process	0.005282495	0.008811202
GO:0009203	ribonucleoside triphosphate catabolic process	0.005282495	0.008811202

GO:0009207	purine ribonucleoside triphosphate catabolic process	0.005282495	0.008811202
GO:0008283	cell proliferation	0.005304051	0.008811909
GO:0009056	catabolic process	0.005458063	0.00892094
GO:0006195	purine nucleotide catabolic process	0.005476645	0.00892094
GO:0009143	nucleoside triphosphate catabolic process	0.005476645	0.00892094
GO:0009154	purine ribonucleotide catabolic process	0.005476645	0.00892094
GO:0009261	ribonucleotide catabolic process	0.005476645	0.00892094
GO:0006325	chromatin organization	0.005535726	0.008982092
GO:0006909	phagocytosis	0.00556945	0.009001786
GO:0033057	multicellular organismal reproductive behavior	0.005880552	0.009406184
GO:1901068	guanosine-containing compound metabolic process	0.005880552	0.009406184
GO:0016477	cell migration	0.005887324	0.009406184
GO:0048585	negative regulation of response to stimulus	0.00630749	0.01003902
GO:0019220	regulation of phosphate metabolic process	0.006600197	0.010425311
GO:0051174	regulation of phosphorus metabolic process	0.006600197	0.010425311
GO:0019438	aromatic compound biosynthetic process	0.006982659	0.010987807
GO:0009968	negative regulation of signal transduction	0.007012385	0.010993099
GO:0018130	heterocycle biosynthetic process	0.007199214	0.01119673
GO:0006152	purine nucleoside catabolic process	0.00722283	0.01119673
GO:0046130	purine ribonucleoside catabolic process	0.00722283	0.01119673
GO:0009166	nucleotide catabolic process	0.007466408	0.011462358
GO:0072523	purine-containing compound catabolic process	0.007466408	0.011462358
GO:0034641	cellular nitrogen compound metabolic process	0.007476646	0.011462358
GO:0048870	cell motility	0.007707711	0.011732888
GO:0031324	negative regulation of cellular metabolic process	0.00770938	0.011732888
GO:0009164	nucleoside catabolic process	0.007971293	0.012000105
GO:0042454	ribonucleoside catabolic process	0.007971293	0.012000105
GO:1901292	nucleoside phosphate catabolic process	0.007971293	0.012000105
GO:0046483	heterocycle metabolic process	0.00804972	0.012074581
GO:0009967	positive regulation of signal transduction	0.00812077	0.012080992
GO:0010558	negative regulation of macromolecule biosynthetic process	0.008140908	0.012080992
GO:2000113	negative regulation of cellular macromolecule biosynthetic process	0.008140908	0.012080992
GO:0035556	intracellular signal transduction	0.008870294	0.013116711
GO:1901658	glycosyl compound catabolic process	0.009054145	0.013341267
GO:0007166	cell surface receptor signaling pathway	0.009247855	0.013578717
GO:0023057	negative regulation of signaling	0.009356069	0.013689406
GO:0051674	localization of cell	0.009529823	0.013894882
GO:0022607	cellular component assembly	0.009663232	0.014040305
GO:0023056	positive regulation of signaling	0.009797491	0.01418595
GO:1901362	organic cyclic compound biosynthetic process	0.010066177	0.014524552
GO:0010648	negative regulation of cell communication	0.010254259	0.014694248
GO:0050790	regulation of catalytic activity	0.010254259	0.014694248

GO:0006725	cellular aromatic compound metabolic process	0.010439307	0.014908188
GO:0006807	nitrogen compound metabolic process	0.010783318	0.015346906
GO:0016568	chromatin modification	0.011196317	0.01588049
GO:0034655	nucleobase-containing compound catabolic process	0.014238225	0.02005858
GO:0048732	gland development	0.014238225	0.02005858
GO:0046578	regulation of Ras protein signal transduction	0.01525164	0.021413918
GO:0001751	compound eye photoreceptor cell differentiation	0.015664752	0.021846828
GO:0051056	regulation of small GTPase mediated signal transduction	0.015664752	0.021846828
GO:0051172	negative regulation of nitrogen compound metabolic process	0.016154762	0.022455119
GO:0007606	sensory perception of chemical stimulus	0.016379648	0.022692071
GO:1901360	organic cyclic compound metabolic process	0.016575366	0.022887178
GO:0071310	cellular response to organic substance	0.017394864	0.023939466
GO:0019439	aromatic compound catabolic process	0.018277049	0.024948339
GO:0044270	cellular nitrogen compound catabolic process	0.018277049	0.024948339
GO:0001754	eye photoreceptor cell differentiation	0.018307414	0.024948339
GO:0019953	sexual reproduction	0.018931532	0.025714817
GO:0046700	heterocycle catabolic process	0.019089848	0.025845671
GO:0022414	reproductive process	0.021564421	0.029101501
GO:1901361	organic cyclic compound catabolic process	0.021680083	0.029163208
GO:0044703	multi-organism reproductive process	0.021989539	0.029484366
GO:0044085	cellular component biogenesis	0.022627153	0.030242061
GO:0007431	salivary gland development	0.022820853	0.030306675
GO:0035272	exocrine system development	0.022820853	0.030306675
GO:0044248	cellular catabolic process	0.023380519	0.030951354
GO:0009150	purine ribonucleotide metabolic process	0.025242295	0.033310244
GO:0045892	negative regulation of transcription, DNA-templated	0.025867455	0.033920531
GO:1902679	negative regulation of RNA biosynthetic process	0.025867455	0.033920531
GO:0032504	multicellular organism reproduction	0.027064677	0.035276857
GO:0006163	purine nucleotide metabolic process	0.027070969	0.035276857
GO:0009259	ribonucleotide metabolic process	0.027542188	0.035667989
GO:0019693	ribose phosphate metabolic process	0.027542188	0.035667989
GO:0009790	embryo development	0.028044015	0.036205431
GO:0048609	multicellular organismal reproductive process	0.028477497	0.036651593
GO:0048729	tissue morphogenesis	0.028862225	0.037032455
GO:0009892	negative regulation of metabolic process	0.029162007	0.037302322
GO:0003002	regionalization	0.029733097	0.037916518
GO:0051253	negative regulation of RNA metabolic process	0.03021757	0.03841685
GO:0046530	photoreceptor cell differentiation	0.031239312	0.039595116
GO:0034645	cellular macromolecule biosynthetic process	0.034638033	0.043769878
GO:0032268	regulation of cellular protein metabolic process	0.035729766	0.045013029
GO:0009059	macromolecule biosynthetic process	0.036408699	0.045730203

GO:0072521	purine-containing compound metabolic process	0.037022744	0.046361814
GO:0045934	negative regulation of nucleobase-containing compound metabolic process	0.037666985	0.047027344
GO:0044710	single-organism metabolic process	0.038802478	0.048300398
GO:0048598	embryonic morphogenesis	0.038992596	0.048392596

Cellular components

ID	Description	pvalue	p.adjust
GO:0005575	cellular_component	5.29E-15	3.12E-13
GO:0031226	intrinsic component of plasma membrane	4.83E-08	1.03E-06
GO:0005576	extracellular region	5.24E-08	1.03E-06
GO:0005887	integral component of plasma membrane	1.58E-07	2.33E-06
GO:0005623	cell	6.83E-07	6.72E-06
GO:0044464	cell part	6.83E-07	6.72E-06
GO:0071944	cell periphery	1.39E-06	1.17E-05
GO:0044459	plasma membrane part	2.02E-06	1.49E-05
GO:0005615	extracellular space	2.35E-06	1.54E-05
GO:0005886	plasma membrane	4.05E-06	2.17E-05
GO:0005911	cell-cell junction	4.05E-06	2.17E-05
GO:0034702	ion channel complex	9.85E-06	4.84E-05
GO:0030424	axon	1.49E-05	6.28E-05
GO:1902495	transmembrane transporter complex	1.49E-05	6.28E-05
GO:0044421	extracellular region part	3.28E-05	0.000129051
GO:0031224	intrinsic component of membrane	3.69E-05	0.000136179
GO:0097458	neuron part	6.63E-05	0.000230038
GO:0016021	integral component of membrane	7.13E-05	0.000233803
GO:0030054	cell junction	0.000134634	0.000418074
GO:0016020	membrane	0.000272115	0.000802738
GO:0044425	membrane part	0.000520431	0.001462162
GO:0045177	apical part of cell	0.000943603	0.00253057
GO:0043005	neuron projection	0.001257608	0.003226037
GO:0043234	protein complex	0.001341416	0.003297647
GO:0005634	nucleus	0.001812013	0.004276351
GO:0005856	cytoskeleton	0.003483126	0.007904017
GO:0044430	cytoskeletal part	0.004527524	0.009893478
GO:0044456	synapse part	0.00483231	0.010182368
GO:0045202	synapse	0.007098626	0.014442032
GO:0042995	cell projection	0.00816187	0.016051678
GO:0043229	intracellular organelle	0.009125999	0.017368837
GO:0000785	chromatin	0.010683642	0.019697965
GO:0043226	organelle	0.012221808	0.021257067
GO:0043231	intracellular membrane-bounded organelle	0.012262613	0.021257067
GO:0043227	membrane-bounded organelle	0.012610125	0.021257067
GO:0005622	intracellular	0.015254498	0.025000427

GO:0005875	microtubule associated complex	0.021788882	0.034744433
GO:0044427	chromosomal part	0.023378246	0.036297802
GO:1990234	transferase complex	0.031659919	0.047895775
GO:0005694	chromosome	0.033790403	0.049840844

Table S4. Fly stocks.

Stock ID	Genotype	Source
30586	*w;; UAS-Psc-IR*	VDRC
39378	*w;; UAS-Su(var)3-9-IR*	VDRC
37715	*w;; UAS-trx-IR*	VDRC
105115	*w;; UAS-nej-IR*	VDRC
22483	*w;; UAS-Sirt6-IR*	VDRC
51282	*w;; UAS-Kr-h1-IR*	VDRC
52486	*w;; UAS-Nipped-A-IR*	VDRC
33459	*w;; UAS-CG2051-IR*	VDRC
	tim-gal4	lab stock
	tim-gal4-UAS-dicer2	lab stock
	W^{1118}	lab stock
	Canton -S	lab stock
169	y[1]	BDSC
807	In(1)w[m4]	BDSC
6599	y[1] w[67c23]	BDSC
13097	y[1] w[67c23]; P{y[+mDint2] w[BR.E.BR]=SUPor-P}*Kr h1*[KG00354]/SM6a	BDSC
16514	y[1]; P{y[+mDint2] w[BR.E.BR]=SUPor-P}*Nipped-A*[KG10162]/CyO	BDSC
20076	y[1] w[67c23]; P{w[+mC] y[+mDint2]=EPgy2}*Psc*[EY06547]/CyO	BDSC
20737	y[1] w[67c23]; P{w[+mC] y[+mDint2]=EPgy2}*trx*[EY12356]/TM3, Sb[1] Ser[1]	BDSC
22496	y[1] w[67c23]; P{w[+mC] y[+mDint2]=EPgy2}*CG2051*[EY21697]	BDSC
6209	In(1)w[m4]; Su(var)3-9[1]/TM3, Sb[1] Ser[1]	BDSC
16514	y[1]; P{y[+mDint2] w[BR.E.BR]=SUPor-P}Nipped-A[KG10162]/CyO	BDSC
20076	y[1] w[67c23]; P{w[+mC] y[+mDint2]=EPgy2}Psc[EY06547]/CyO	BDSC
20737	y[1] w[67c23]; P{w[+mC] y[+mDint2]=EPgy2}trx[EY12356]/TM3, Sb[1] Ser[1]	BDSC
22496	y[1] w[67c23]; P{w[+mC] y[+mDint2]=EPgy2}CG2051[EY21697]	BDSC
6209	In(1)w[m4]; Su(var)3-9[1]/TM3, Sb[1] Ser[1]	BDSC

2971	w;; UAS-Hr38-IR	VDRC
7206	w;; UAS-DopR-IR	VDRC
8933	w;; UAS-Fmr1-IR	VDRC
11963	w;; UAS-CG11155-IR	VDRC
27406	w;; UAS-sif-IR	VDRC
35439	w;; UAS-Thor-IR	VDRC
35877	w;; UAS-Nf1-IR	VDRC
38540	w;; UAS-CG11597-IR	VDRC
39529	w;; UAS-pho-IR	VDRC
42582	w;; UAS-CG7589-IR	VDRC
42660	w;; UAS-Calx-IR	VDRC
46542	w;; UAS-nrv1-IR	VDRC
52268	w;; UAS-mod(mdg4)-IR	VDRC
21719	w;; UAS-sug-IR	VDRC

Table S5. qPCR primers information

Primer name	Sequence 5' --- 3'	Target gene	PCR product size (bp)
Hr38_F	TTTAACGAGCACGTGAGCTG	*Hr38*	131
Hr38_R	TTCGGTTGTGGTAGTTGCAG		
CG3099_F	ACTGGACGCATTCTTTACGC	*CG3099*	107
CG3099_R	ATTGGAGCGACTGATGGAAC		
Psc_F	TGGGGCAGAAAACATAGAGC	*PSc*	103
Psc_R	CTGCACATTCAAACCACACC		
CG2051_F	CAATGATGCCCTGACCTTTC	*CG2051*	137
CG2051_R	AATCGACGCCCAAGTAGATG		
CG11597_F	GCCGTGTCTTCGATCTCTTG	*CG11597*	141
CG11597_R	TTCCACTTTCGGGTATCTCG		
su(var)3-9_F	GGCCCAGCTTAAGTACAACATC	*su(var)3-9*	111
su(var)3-9_R	AAGGATCGGATGACGATCAG		
CG13796_F	TCAGATCGAGATTCGGGTTC	*CG13796*	122
CG13796_R	CATTCACCTGCAAACAGTGC		
rpL32_F	AAGTGTGCGGCTCGTATTTC	*rp49 (rpL32)*	109
rpL32_R	ATTGAGTTTCCGGTGTGTCG		
hep_F	CCATGTGCTTTGACAAGCTG	*hep*	148
hep_R	ATGTTCGAGGGCTTCACATC		
Sirt6_F	TTCCTTCGATGAAGCCAGAC	*Sirt6*	118
Sirt6_R	TCCGGATTTCAAGTGGAGAC		
sug_F	TCATCACCTGTACGGCAAAC	*sug*	141
sug_R	AGCAAAGCCATCCAGAACAC		
per_F	ACTGGAACAGGCAATGGAAC	*per*	134
per_R	TTCTCCATCTCGTCGTTGTG		
Kr-h1_F	CGTCGAACTTTTCGGTTCTC	*Kr-h1*	120
Kr-h1_R	GCTGCTGGGCATTAACTTTC		
Thor_F	CAGCAACTGCCAAATCCAAC	*Thor*	132
Thor_R	GGGTCAATATGACCGAGAGAAC		
So_F	ATGCCATTATAGCCCCACAC	*so*	100
So_R	ATCCTTCCAGGAGATTGTGC		
Yp1_F	ACATGCAGCGCTACAATCTG	*Yp1*	148
Yp1_R	TGGGTCTTGGCGTTCTTAAC		
Fmr1_F	GGCGGAGAATGTCAAAAAGG	*Fmr1*	134
Fmr1_R	TTACTCCGTCGAACGATGTG		

Fig. S1. Pairwise expression correlation among sample replicates.

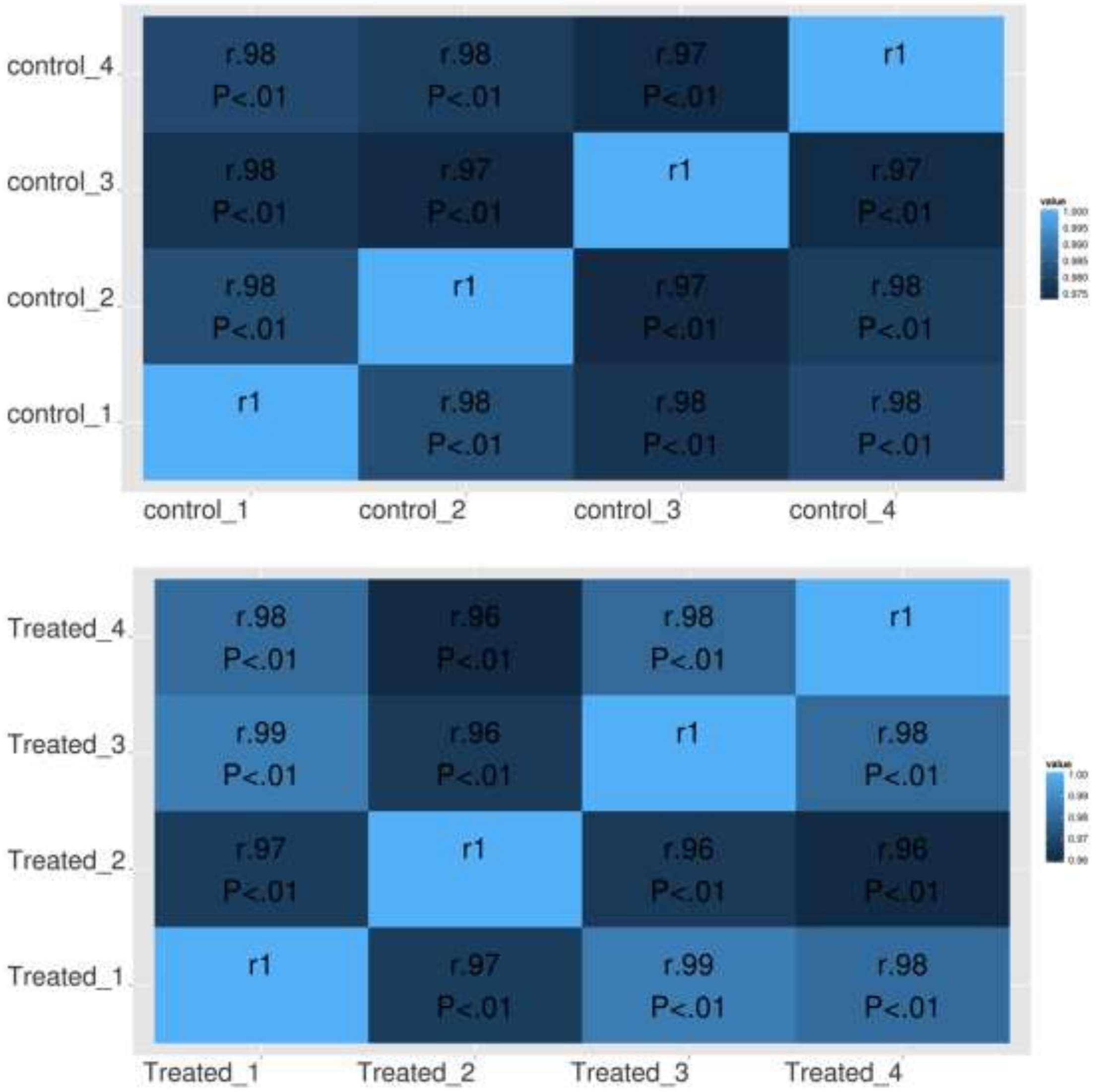

Fig S2. **Enriched biological processes (BP) among DEG.** The analysis was carried by clusterProfiler gene ontology Bioconductor package. The adjusted P-value is color-coded as indicated in the key at the top.

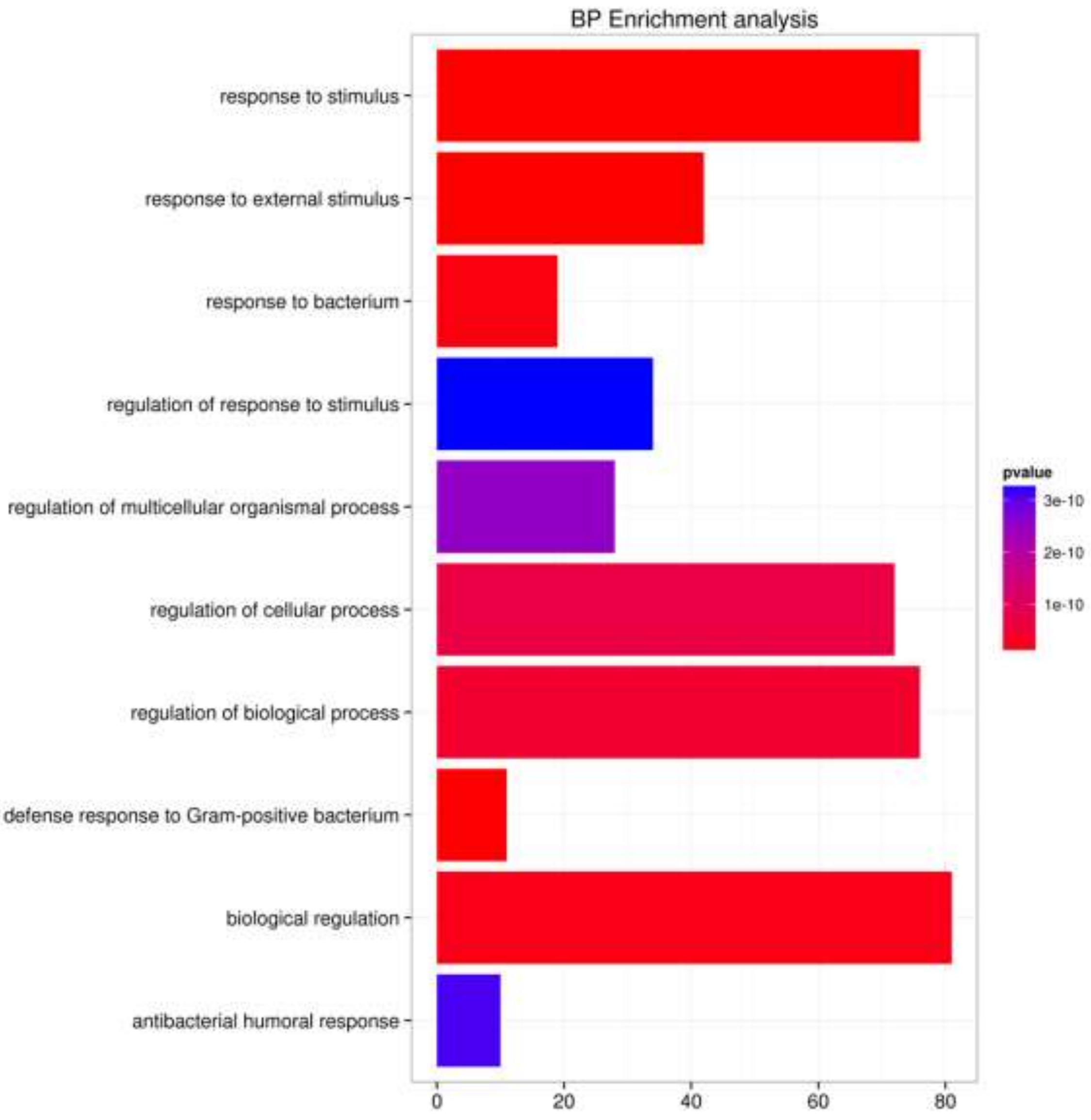

Fig S3. Enriched molecular functions (MF) among DEG.

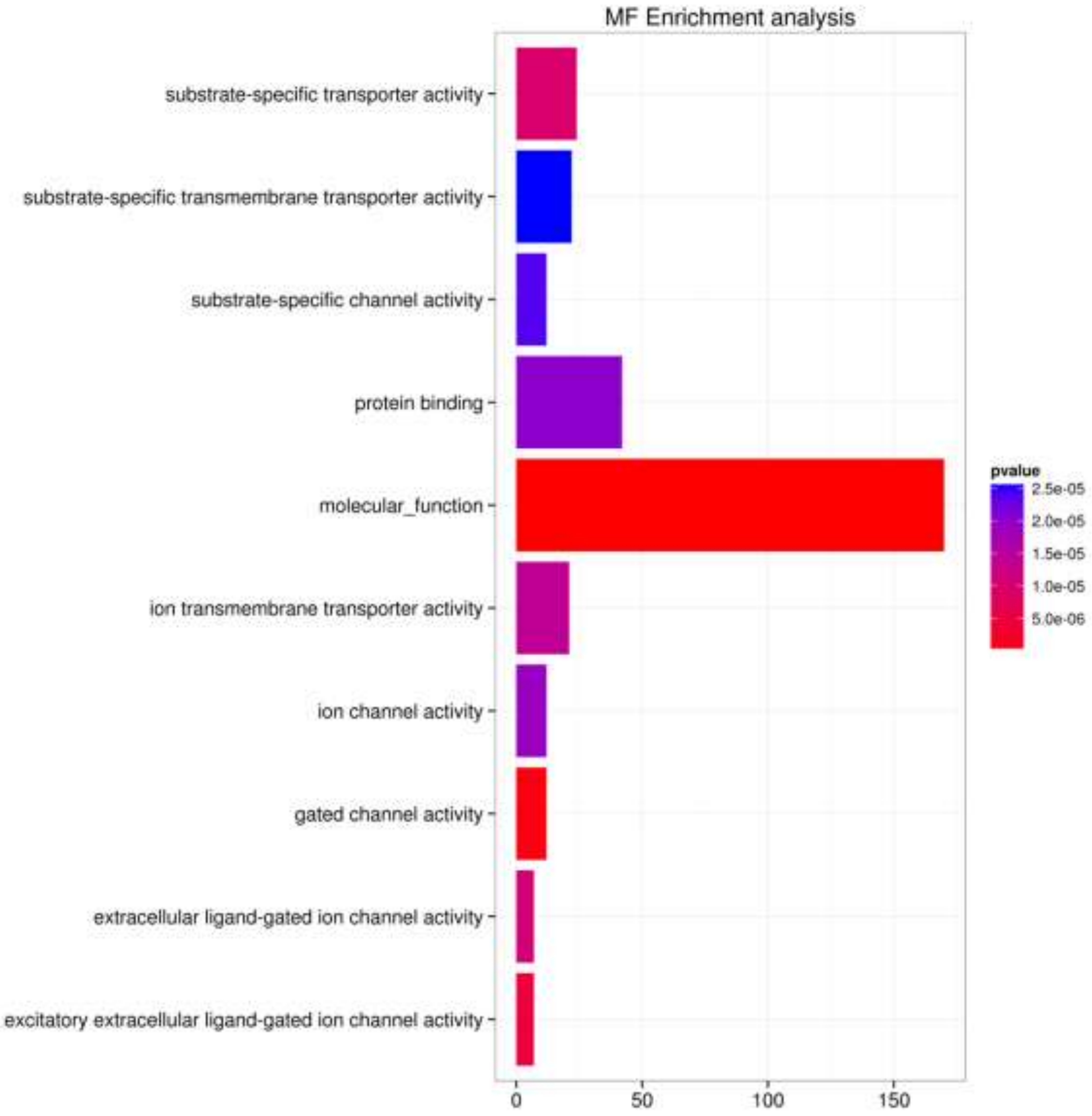

Fig S4. Enriched cellular components (CC) among DEG. clusterProfiler gene ontology analysis.

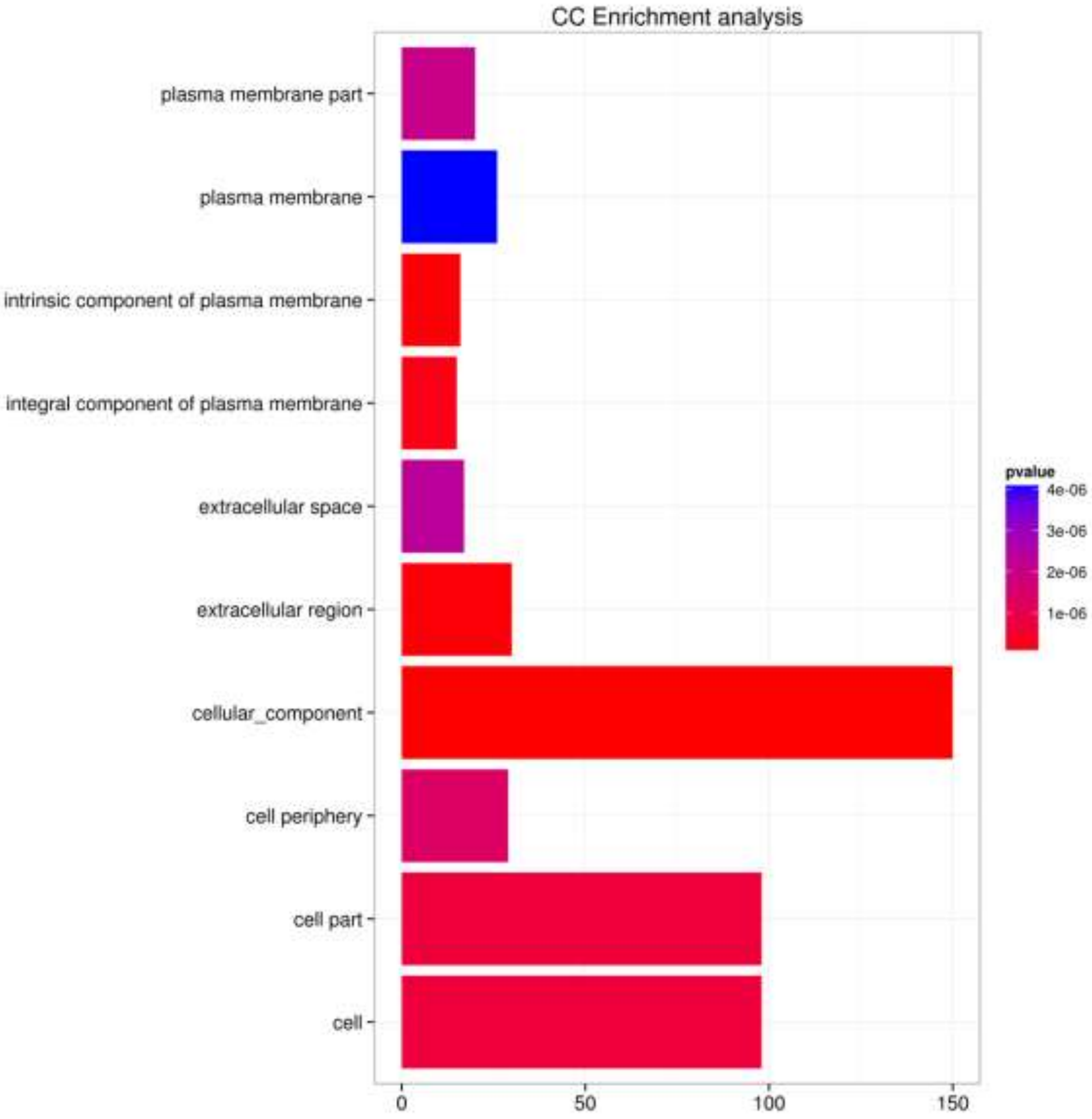

Fig S5. **Activity profiles of Nipped-A ^{KG10162} and Psc^{EY06547} mutants.** Activity profile of Nipped-A ^{KG10162} (middle), Psc^{EY06547} (bottom) and their background control (top panel). Flies under LD and DD conditions. Each plot shows average data from 32 flies for each genotype.

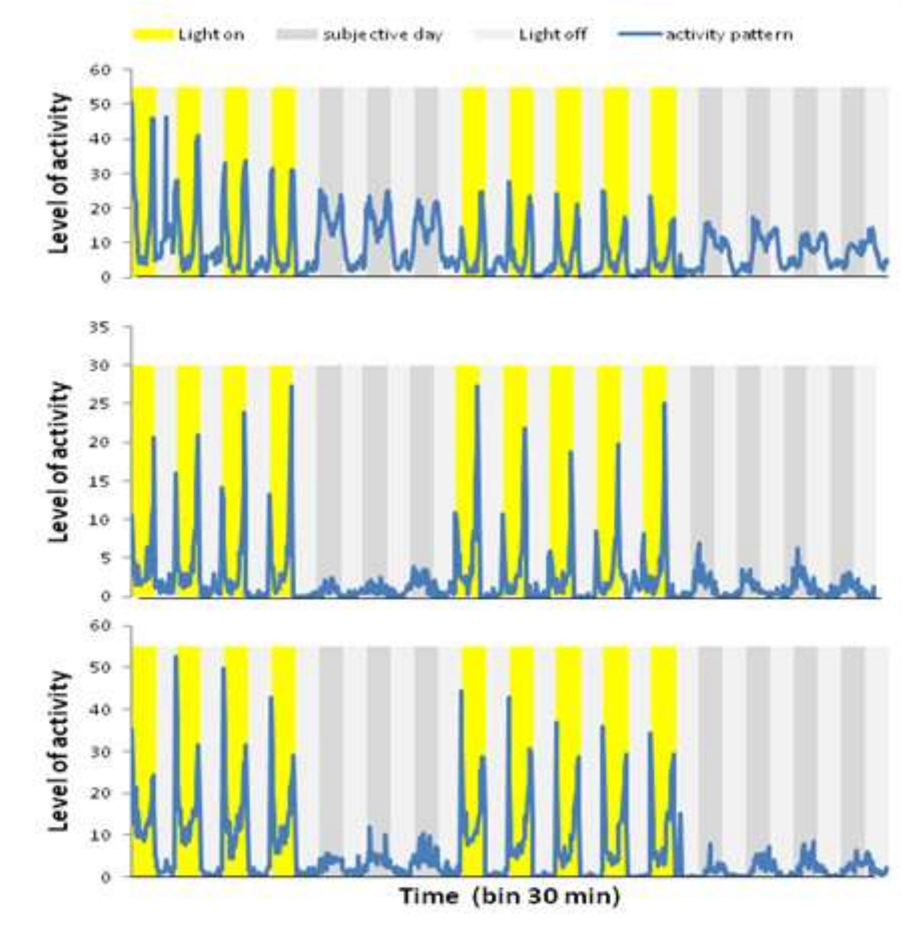

Supplement: Additional file 1: Table S1. — List of differentially expressed genes (DEGs). Table S2. Validation of differentially expressed genes identified via microarray analysis by qPCR. Table S3. Gene Ontology (GO) categories enrichment. Table S4. Fly stocks. Table S5. qPCR primers information. Figure S1. Pairwise correlation among sample replicates. Figure S2. Enriched biological processes (BP) among DEG as reported by the clusterProfiler gene ontology analysis. The adjusted P value is color-coded as indicated in the key at the top. Figure S3. Enriched molecular functions (MF) among DEG as reported by the clusterProfiler gene ontology analysis. The adjusted P value is color-coded as indicated in the key at the top. Figure S4. Enriched cellular components (CC) among DEG as reported by the clusterProfiler gene ontology analysis. Figure S5. Activity profiles of Nipped-A KG10162 and PscEY06547 mutants. (PDF 665 kb) [file 12864_2015_1787_MOESM1_ESM.pdf]
